# Using genetically incorporated unnatural amino acids to control protein functions in mammalian cells

**DOI:** 10.1042/EBC20180042

**Published:** 2019-05-15

**Authors:** Alexander R. Nödling, Luke A. Spear, Thomas L. Williams, Louis Y.P. Luk, Yu-Hsuan Tsai

**Affiliations:** School of Chemistry, Cardiff University, Cardiff, Wales, United Kingdom

**Keywords:** chemical biology, genetic code expansion, protein chemistry, protein engineering, unnatural amino acid

## Abstract

Genetic code expansion allows unnatural (non-canonical) amino acid incorporation into proteins of interest by repurposing the cellular translation machinery. The development of this technique has enabled site-specific incorporation of many structurally and chemically diverse amino acids, facilitating a plethora of applications, including protein imaging, engineering, mechanistic and structural investigations, and functional regulation. Particularly, genetic code expansion provides great tools to study mammalian proteins, of which dysregulations often have important implications in health. In recent years, a series of methods has been developed to modulate protein function through genetically incorporated unnatural amino acids. In this review, we will first discuss the basic concept of genetic code expansion and give an up-to-date list of amino acids that can be incorporated into proteins in mammalian cells. We then focus on the use of unnatural amino acids to activate, inhibit, or reversibly modulate protein function by translational, optical or chemical control. The features of each approach will also be highlighted.

## Introduction

Knowledge of protein function is of pivotal importance to life science research. It can guide conventional drug development programmes and lead to novel strategies to address currently non-targetable systems [1–3]. In order to understand the precise role and interacting network of a protein, it is essential to analyse it within its native environment. For a mammalian protein, its function often also depends on its host cell (e.g. cell type and cell cycle stage), specific subcellular location and post-translational modifications. In addition, a protein of interest often exists in the presence of other closely related homologues (e.g. proteins within the same family), making it difficult to decipher the precise function of a specific protein in cells. Targeting the protein by small-molecule inhibition is often not possible in these cases, as protein homologues will also be affected. To tackle this problem, over the last two decades there has been a drive to develop and refine the technique of genetic code expansion which allows researchers to exploit the cellular translation machinery for site-specific incorporation of unnatural (non-canonical) amino acids into target proteins [4–14]. Consequently, this enables the use of building blocks beyond the 20 canonical amino acids and incorporation of unnatural amino acids with unprecedented functionality into target proteins in live cells. The repurposing of the translational machinery by this approach has paved the way for revealing the functions of proteins under physiological conditions [15–19]. For example, the technique can be used to site-specifically introduce an unnatural amino acid into the homologue of interest, whereby unique functionality (on the unnatural amino acid) can be used for selective activation, inhibition, or reversible regulation of the target homologue [7].

**Figure 1:**
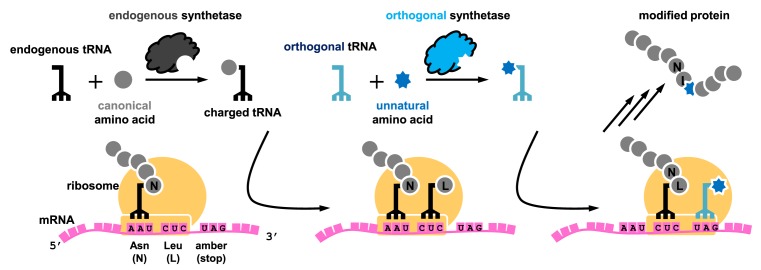
Mechanism of genetic code expansion for site-specific incorporation of an unnatural amino acid by amber suppression

At the molecular level, the mechanism of protein translation is highly conserved in all organisms, where the cellular machinery ‘translates’ every nucleotide triplet as a codon consecutively on the mRNA into the corresponding amino acid. In nature, the endogenous aminoacyl-tRNA synthetase (aaRS)/tRNA pairs within the cell decode 61 of the total 64 codons to 20 canonical amino acids. The remaining three codons (UAG, UGA and UAA) are used for translation termination, and hence they are also known as ‘stop’ codons. In order to achieve site-specific incorporation of an unnatural amino acid, an orthogonal aaRS/tRNA pair is needed, which must decode a codon that does not correspond to any canonical amino acid, a so-called blank codon (Figure 1). Stop codons are most commonly used as a blank codon in genetic code expansion, and decoding of a stop codon is known as ‘suppression’ because it suppresses the translation termination. The amber stop codon (UAG) is often used as the blank codon due to its minimal occurrence in most organisms.

**Figure 2:**
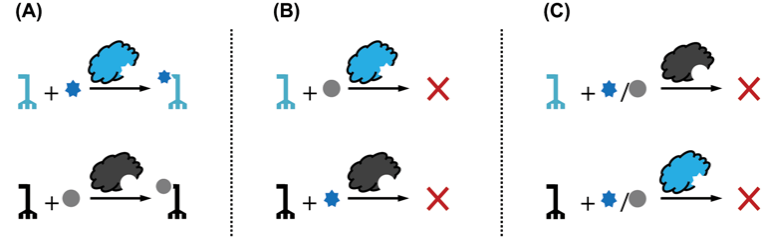
Allowed and not allowed reactivities between the orthogonal and endogenous aaRS/tRNA pairs (**A**) Matching amino acid and aaRS/tRNA pairs; (**B**) mismatched amino acids; (**C**) mismatched aaRS/tRNA pairs.

Within the concept of genetic code expansion, ‘orthogonality’ refers to the non-reactivity of the orthogonal aaRS/tRNA pair with the endogenous pair and canonical amino acids in the host cell. The orthogonal synthetase must only acylate the orthogonal tRNA with the designated unnatural amino acid; neither canonical amino acids nor endogenous tRNAs are substrates of the orthogonal synthetase; similarly, neither the unnatural amino acid nor orthogonal tRNA is a substrate of the endogenous synthetases (Figure 2).

Besides the amber codon, other stop codons [20–26] and different four-nucleotide codons [27,28] have been used as a blank codon. The use of four-nucleotide codons expands the theoretical codon numbers from 4^3^ (64) to 4^4^ (256) so that multiple different unnatural amino acids can be incorporated at the same time. However, decoding a four-nucleotide codon by the ribosome is less efficient than decoding the normal three-nucleotide codons. Although this issue has been addressed in *Escherichia coli* through ribosome engineering [29–31], the lower efficiency in decoding four-nucleotide codons remains an issue in mammalian systems [27,28].

To date, many unnatural amino acids (**1**–**110**, Table 1) can be site-specifically incorporated into proteins produced by mammalian cells using genetic code expansion [5,32]. While the amino acids are structurally diverse, the majority of them can be incorporated through only a few orthogonal synthetases and their mutants. The Pyrrolysyl-tRNA synthetase (PylRS)/tRNA^Pyl^ pairs from archaea species *Methanosarcina barkeri* (*Mb*) and *Methanosarcina mazei* (*Mm*) have proven to be extraordinarily useful pairs [4]. The tRNA^Pyl^ naturally recognises the UAG codon and thus engineering of this tRNA is not needed. In addition, this pair is orthogonal in both *E. coli* and mammalian cells; hence, it facilitates the engineering of PylRS in *E. coli* and subsequently using the engineered PylRS mutant for incorporation of the designated unnatural amino acid in mammalian systems. As shown in Table 1, a wide range of amino acids has been incorporated into proteins in mammalian cells through only a few point mutations on the *PylRS* gene.

**Table 1:** Overview of unnatural amino acids that have been successfully incorporated into proteins in mammalian cells and used for a variety of applications to date

Amino acid	aaRS	Mutations	tRNA	Application
**Cysteine and selenocysteine derivatives**				
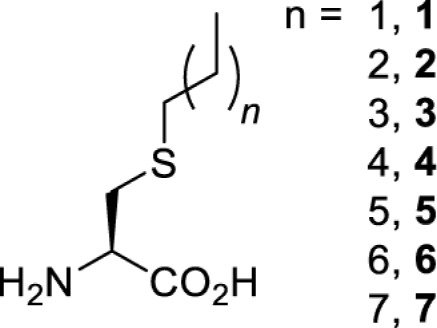	*Ec*LeuRS [24]	M40I T252A Y499I Y527A H529G	${EctRNA}_{CUA}^{Leu}$	Method development
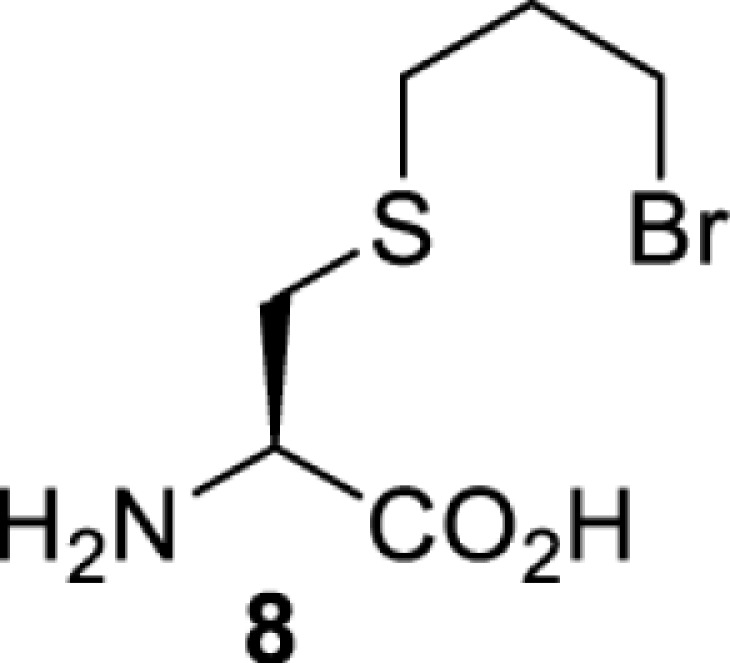	*Ec*LeuRS [24]	M40I T252A Y499I Y527A H529G	${EctRNA}_{CUA}^{Leu}$	Method development
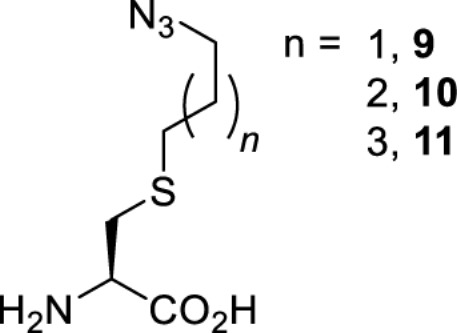	*Ec*LeuRS [24]	M40I T252A Y499I Y527A H529G	${EctRNA}_{CUA}^{Leu}$	Method development
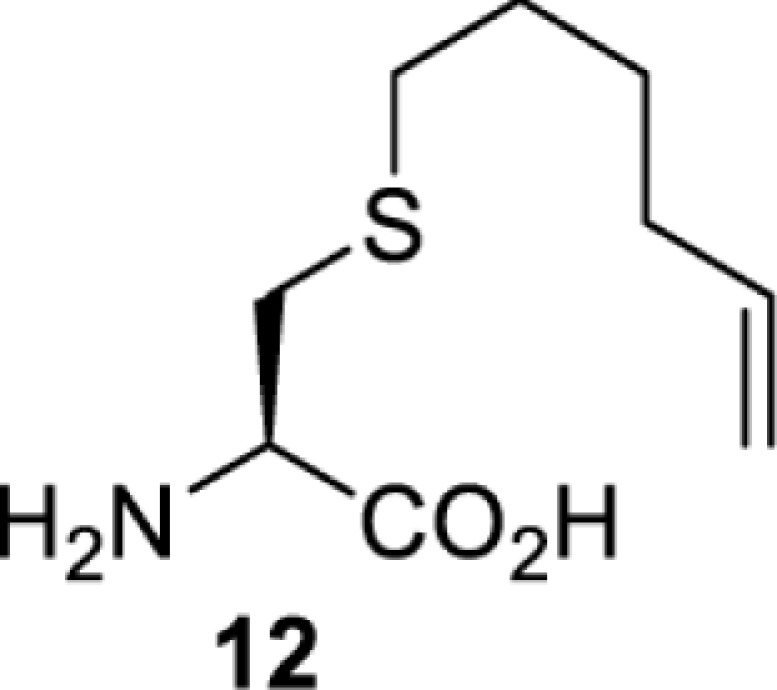	*Ec*LeuRS [24]	M40I T252A Y499I Y527A H529G	${EctRNA}_{CUA}^{Leu}$	Method development
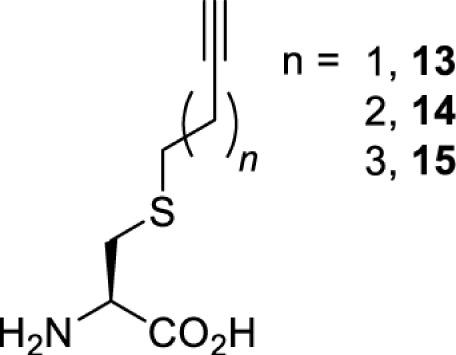	*Ec*LeuRS [24]	M40I T252A Y499I Y527A H529G	${EctRNA}_{CUA}^{Leu}$	Method development
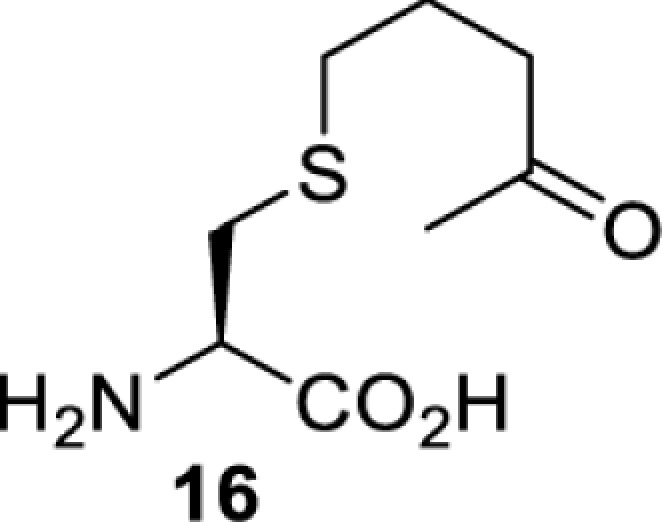	*Ec*LeuRS [24]	M40I T252A Y499I Y527A H529G	${EctRNA}_{CUA}^{Leu}$	Method development
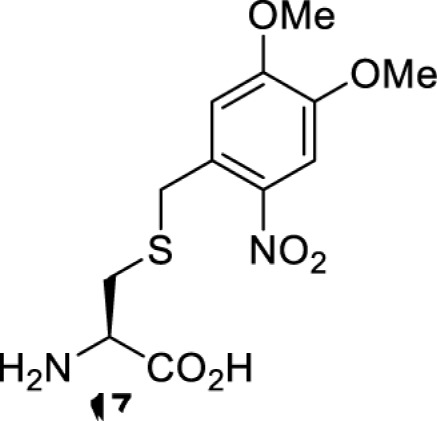	*Ec*LeuRS [71]	M40G L41Q T252A Y499L Y527G H537F	${EctRNA}_{CUA}^{Leu}$	Photoactivation
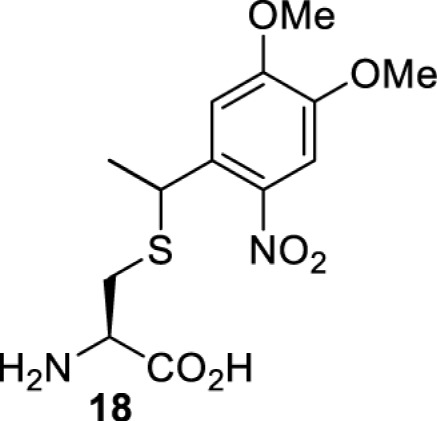	*Ec*LeuRS [72]	M40G L41Q Y499L Y527G H537F	${EctRNA}_{CUA}^{Leu}$	Photoactivation
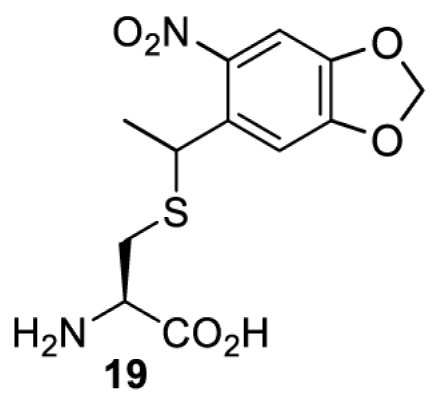	*Mb*PylRS [73,74]	N311Q C313A V366M	${MbtRNA}_{CUA}^{Pyl}$	Photoactivation
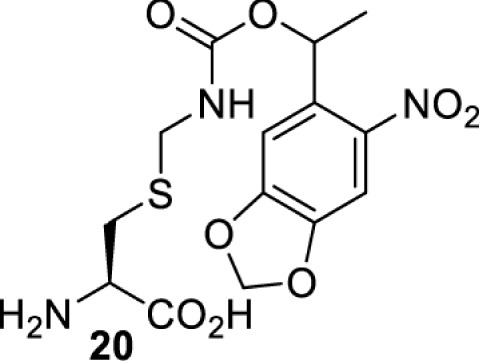	*Mb*PylRS [75]	M241F A267S Y271C L274M	${MbtRNA}_{CUA}^{Pyl}$	Photoactivation
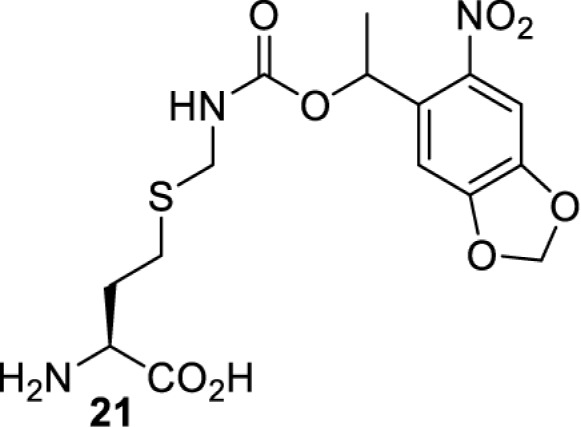	*Mb*PylRS [75]	M241F A267S Y271C L274M	${MbtRNA}_{CUA}^{Pyl}$	Photoactivation
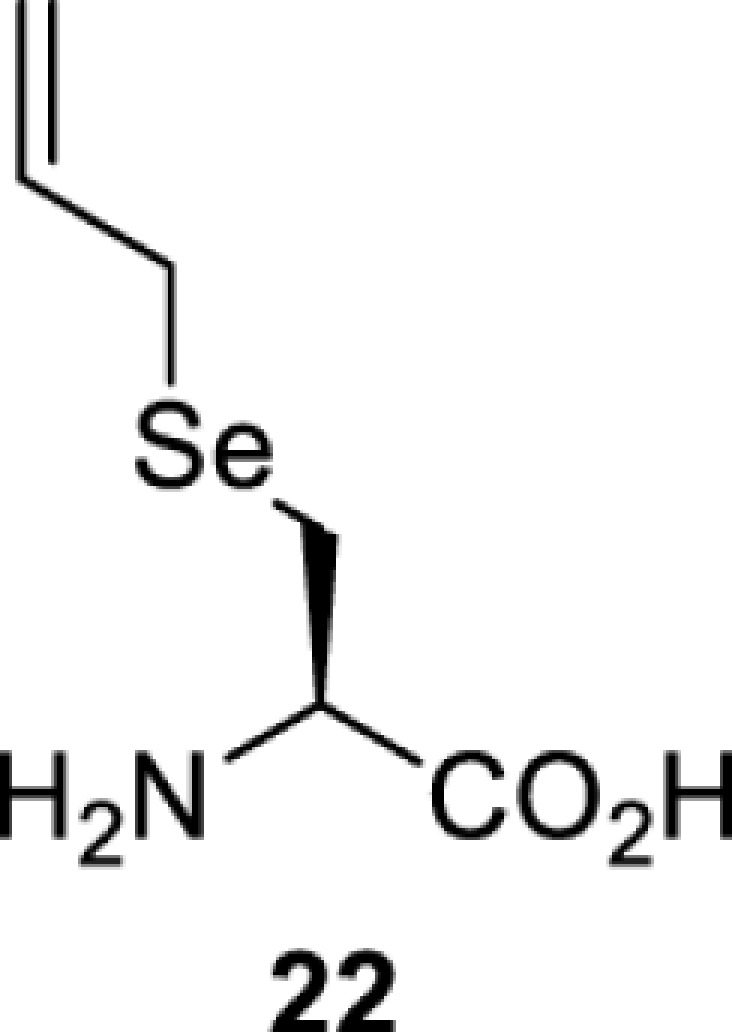	*Mb*PylRS [76]	C313W W382T	MbPyltRNA	Method development
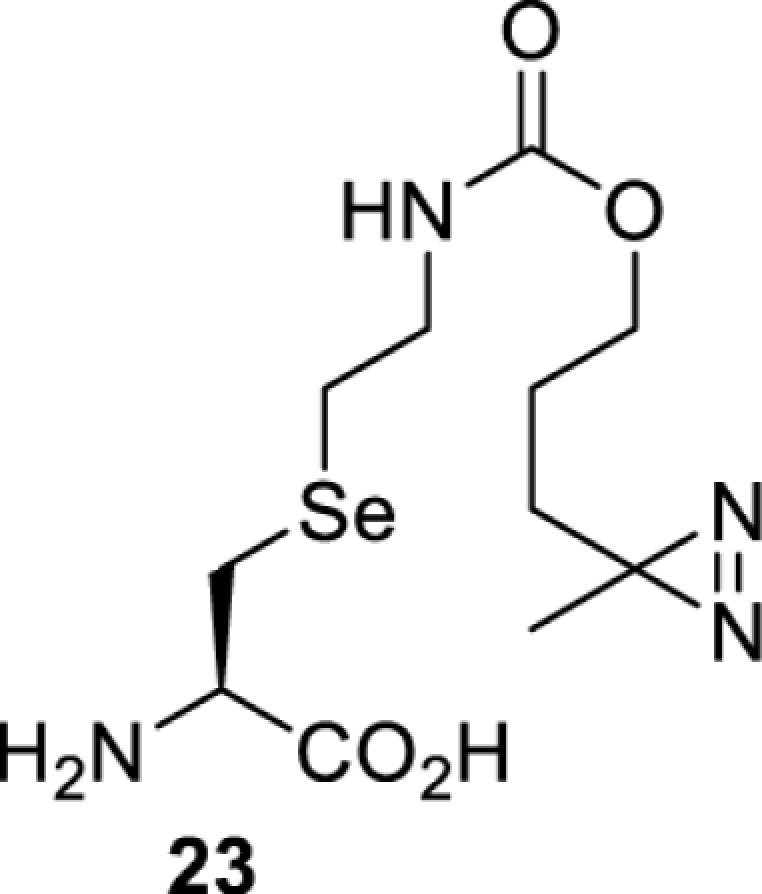	*Mb*PylRS [40]	L274A C313S Y349F	${MbtRNA}_{CUA}^{Pyl}$	Photocrosslinking
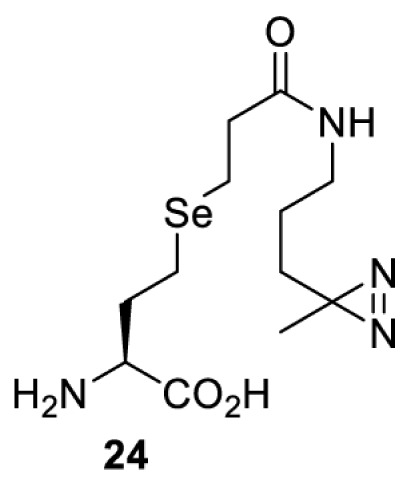	*Mb*PylRS [40]	L274A C313S Y349F	${MbtRNA}_{CUA}^{Pyl}$	Photocrosslinking
**Phenylalanine derivatives**				
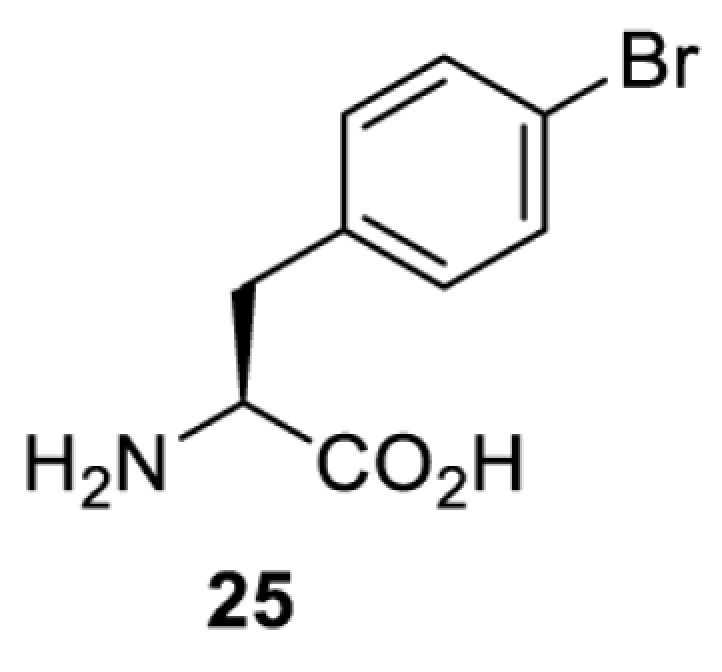	*Ec*TyrRS [77]	Y37V D182S F183M D265R	${EctRNA}_{CUA}^{Tyr}$	Method development
			${BstRNA}_{CUA}^{Tyr}$	
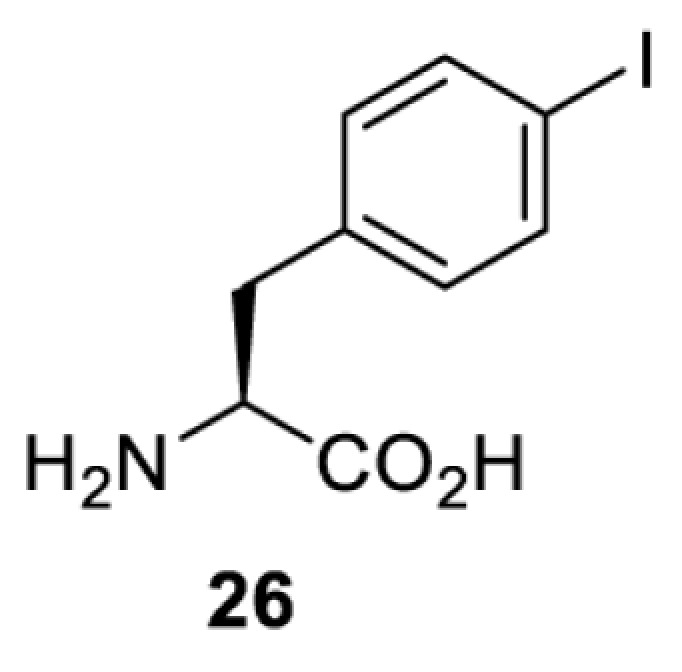	*Ec*TyrRS [35,37,43,77]	Y37V D182S F183M D265R [77] Y37I D182S F183M [37,43] Y37V D165G D182S F183M L186A D265R [35]	${EctRNA}_{CUA}^{Tyr}$ [35,77]	Method development [35,37,77] Protein engineering [43]
			${BstRNA}_{CUA}^{Tyr}$ [37,43,77]	
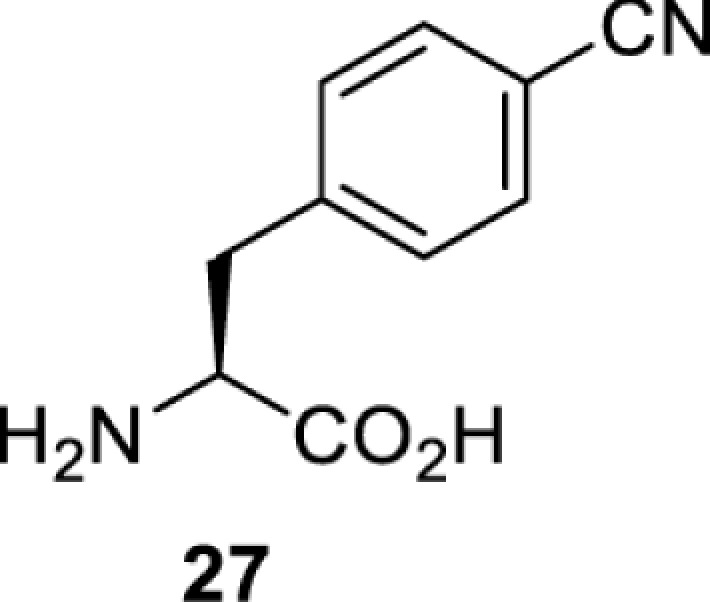	*Ec*TyrRS [77]	Y37V D182S F183M D265R	${EctRNA}_{CUA}^{Tyr}$	Method development
			${BstRNA}_{CUA}^{Tyr}$	
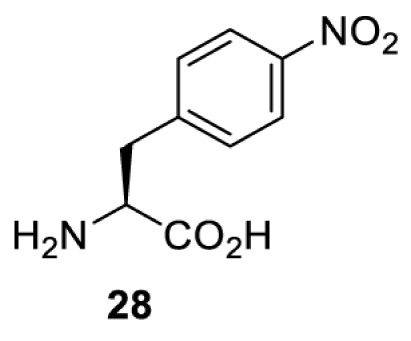	*Ec*TyrRS [77]	Y37V D182S F183M D265R	${EctRNA}_{CUA}^{Tyr}$	Method development
			${BstRNA}_{CUA}^{Tyr}$	
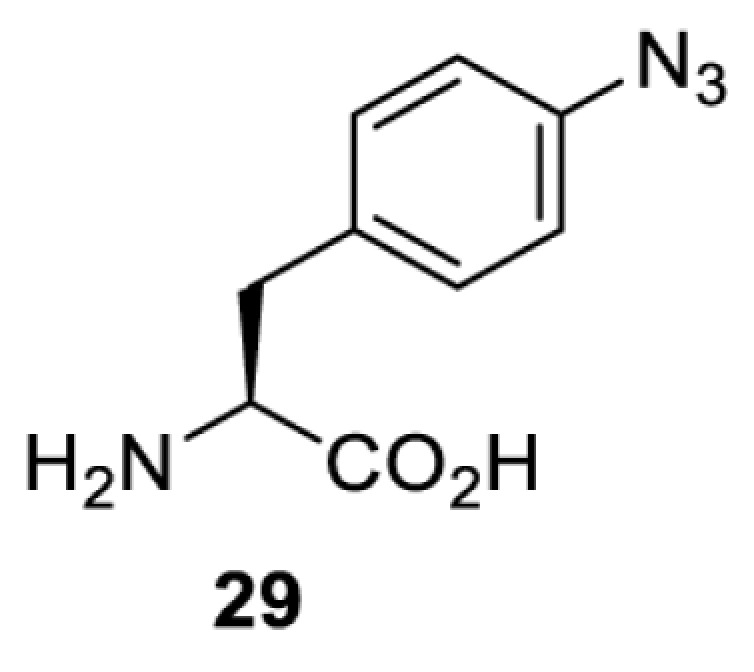	*Ec*TyrRS_CUA_ [16,17,35,37,38,43,49,55,68,77–96]	Y37L D182S F183A L186A D265R [78,81,84,85] Y37V D182S F183M D265R [77,90] Y37L D182S F183M L186A [16,17,37,38,43,49,55,68,79,80,82,83,86–89,91–96] Y37V D165G D182S F183M L186A D265R [35]	${EctRNA}_{CUA}^{Tyr}$ [35,77,78,81,86,90]	Bioorthogonal labelling [38,79,83,87–89,96] Method development [17,25,35,37,77,78,90] Photocrosslinking [38,68,81,84,85,91–95] Protein engineering [43,49,55,83] Spectroscopic probe [16,80,82,86]
			${BstRNA}_{CUA}^{Tyr}$ [16,17,37,38,43,49,55,68,77,79,80,82–85,87–96]	
	*Ec*TyrRS_UCA_ [25]	Y37V D182S F183M	${EctRNA}_{UCA}^{Tyr}$	
			${BstRNA}_{UCA}^{Tyr}$	
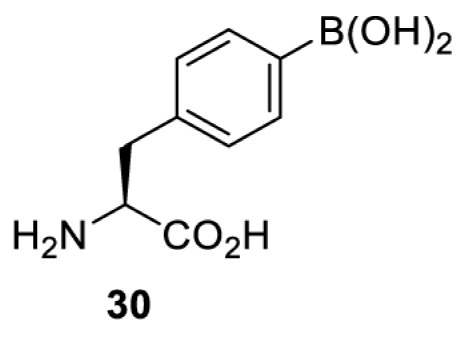	*Ec*TyrRS [35,97]	Y37I D182S F183M D265R [97] Y37S D182S F183A L186E D265R [35] Y37G D182S F183I L186E D265R [35] Y37S D182S F183I L186E D265R [35]	${EctRNA}_{CUA}^{Tyr}$ [35,97]	Method development [35] Spectroscopic probe [97]
			${BstRNA}_{CUA}^{Tyr}$ [97]	
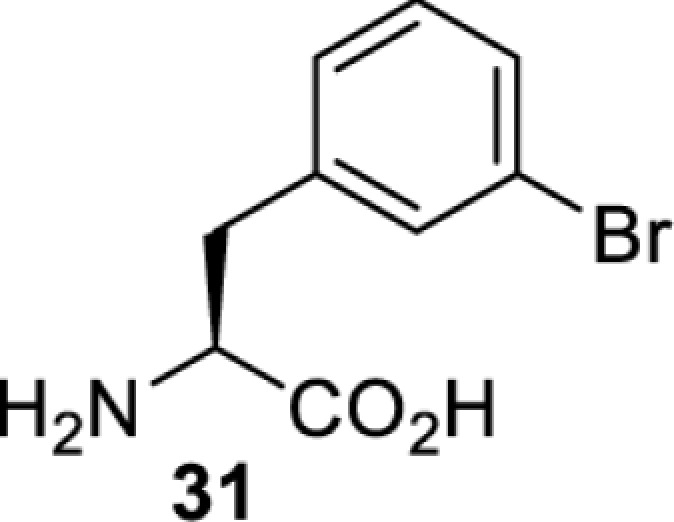	*Mm*PylRS [50]	L301M Y306L L309A C348F	${MmtRNA}_{CUA}^{Pyl}$	Method development
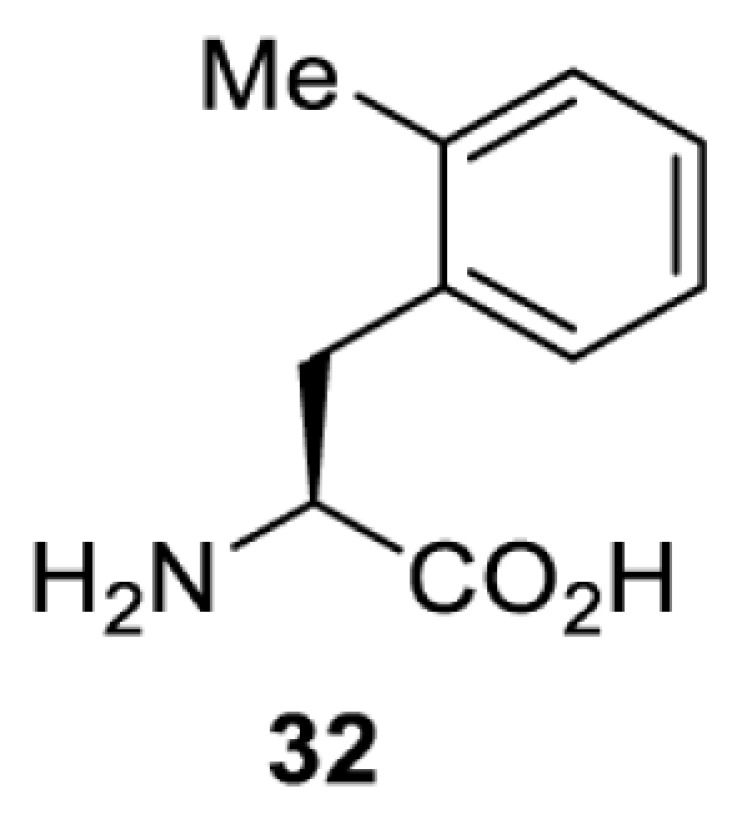	*Mm*PylRS [98]	N346A C348A	${MmtRNA}_{CUA}^{Pyl}$	Method development
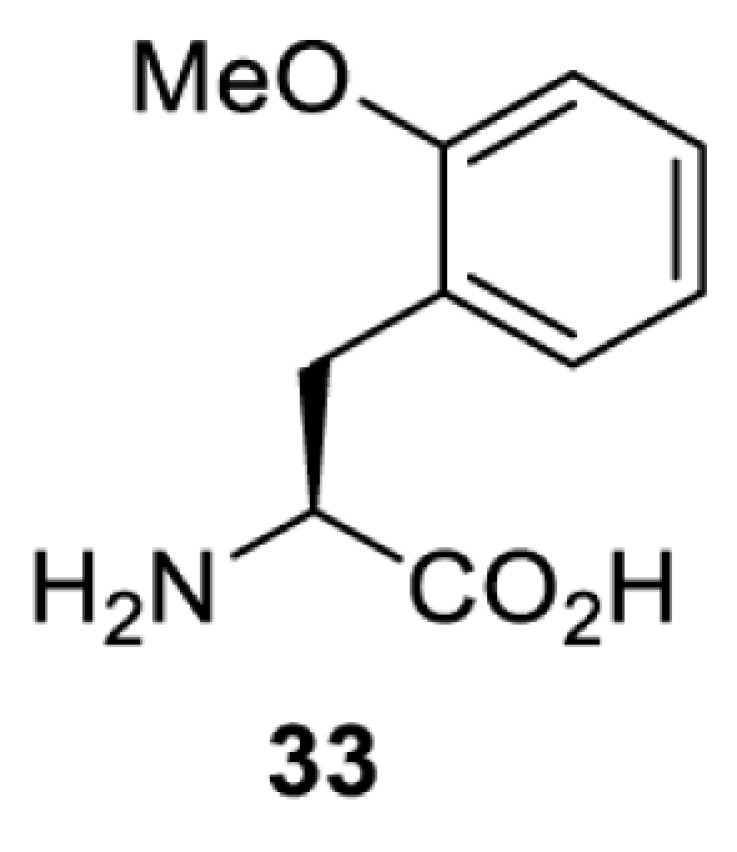	*Mm*PylRS [98]	N346A C348A	${MmtRNA}_{CUA}^{Pyl}$	Method development
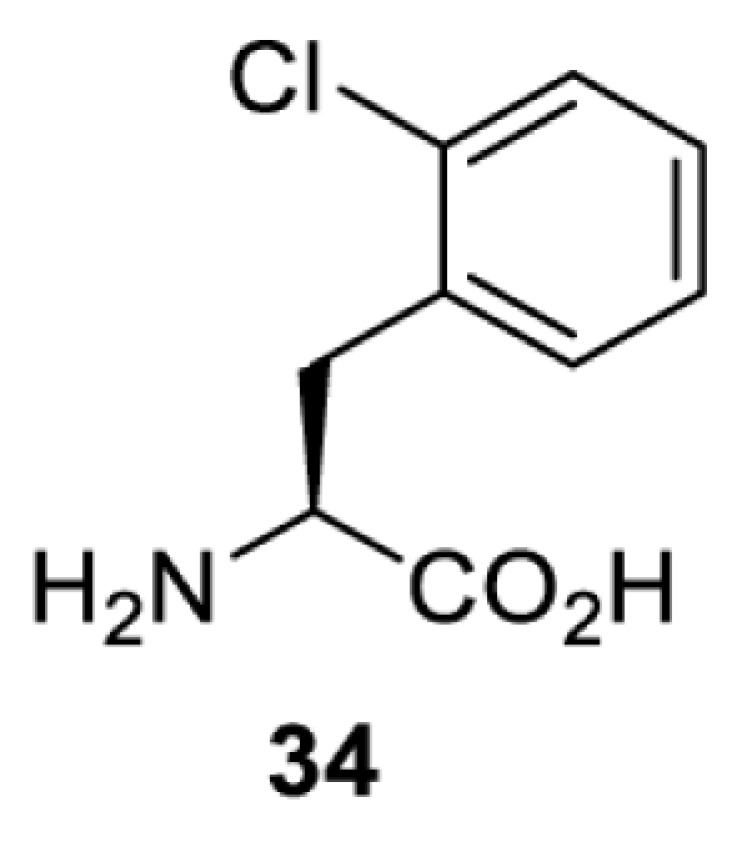	*Mm*PylRS [98]	N346A C348A	${MmtRNA}_{CUA}^{Pyl}$	Method development
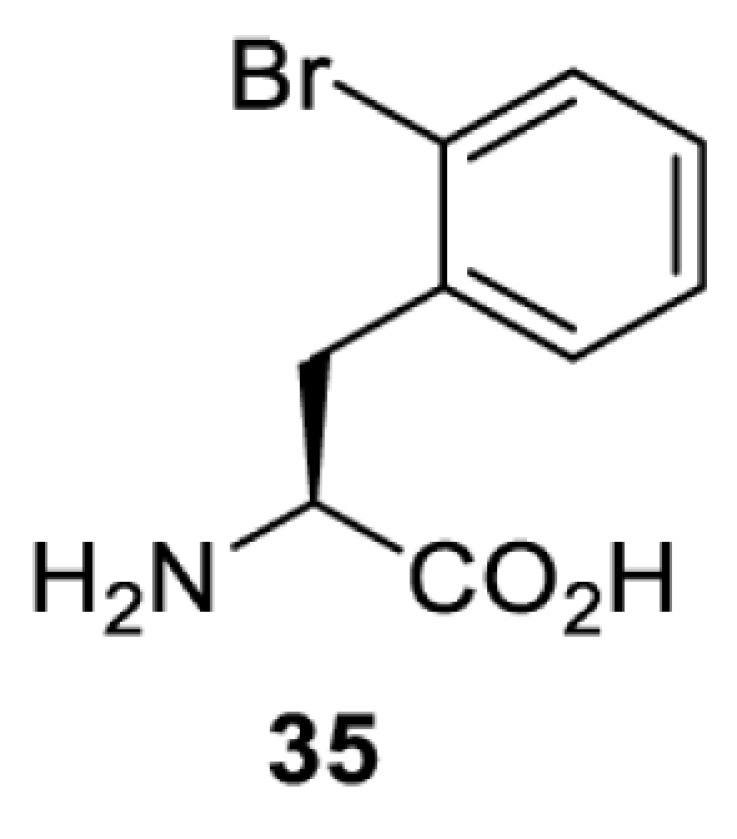	*Mm*PylRS [98]	N346A C348A	${MmtRNA}_{CUA}^{Pyl}$	Method development
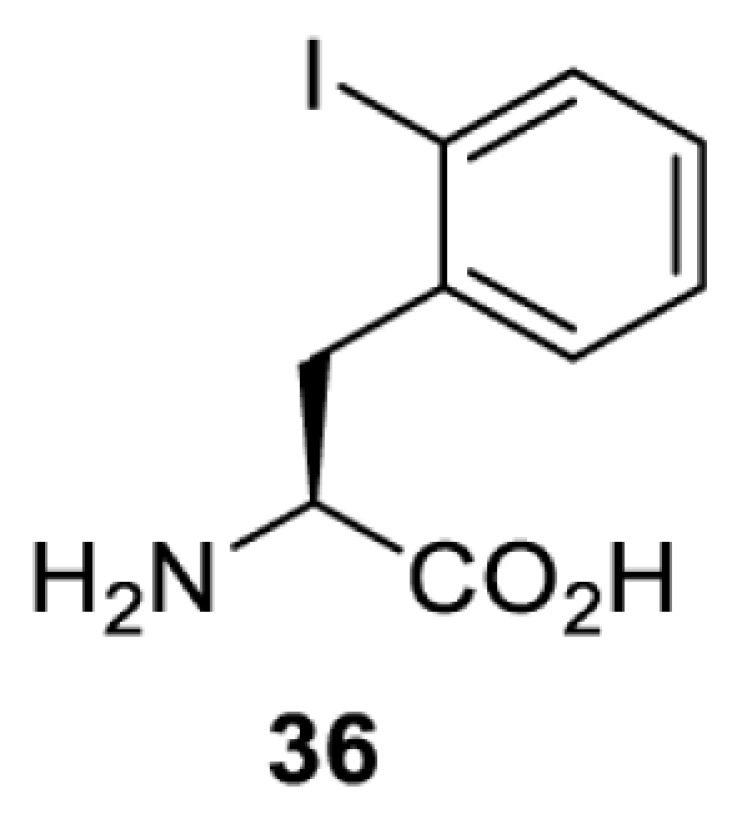	*Mm*PylRS [98]	N346A C348A	${MmtRNA}_{CUA}^{Pyl}$	Method development
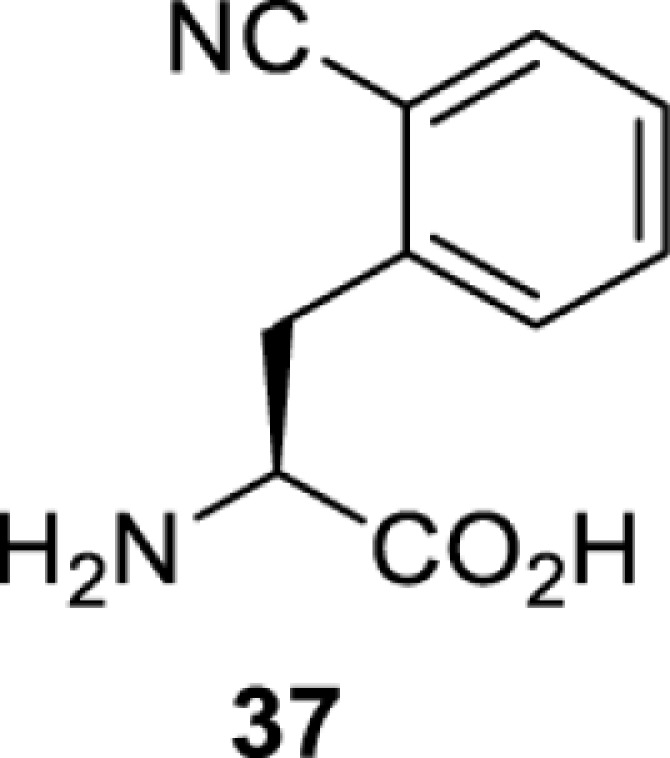	*Mm*PylRS [98]	N346A C348A	${MmtRNA}_{CUA}^{Pyl}$	Method development
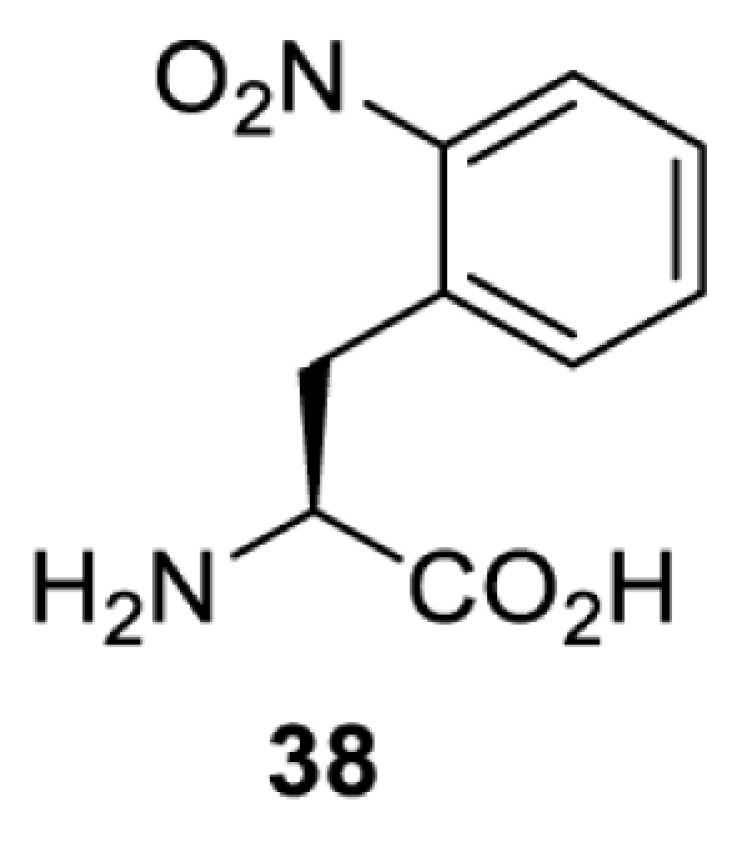	*Mm*PylRS [98]	N346A C348A	${MmtRNA}_{CUA}^{Pyl}$	Method development
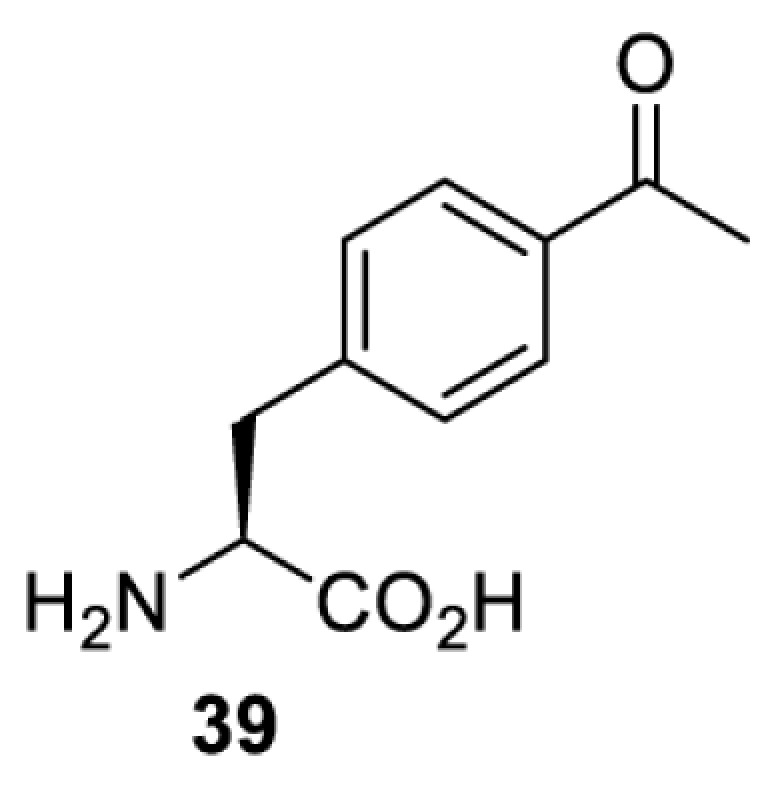	*Ec*TyrRS_CUA_ [21,35,37,43,77,83,87,90,99,100]	Y37I N165G D182G F183M L186A [83,99] Y37I D182G F183M L186A [37,43,87,100] Y37V D182S F183M D265R [21,77,90] Y37V D165G D182S F183M L186A D265R [35]	${EctRNA}_{CUA}^{Tyr}$ [21,35,77,90]	Bioorthogonal labelling [83,87,100] Method development [25,35,37,77,83,90,99] Protein engineering [21,43]
			${BstRNA}_{CUA}^{Tyr}$ [37,43,77,83,87,99,100]	
	*Ec*TyrRS_UCA_ [25]	Y37V D182S F183M	${EctRNA}_{UCA}^{Tyr}$	
			${BstRNA}_{UCA}^{Tyr}$	
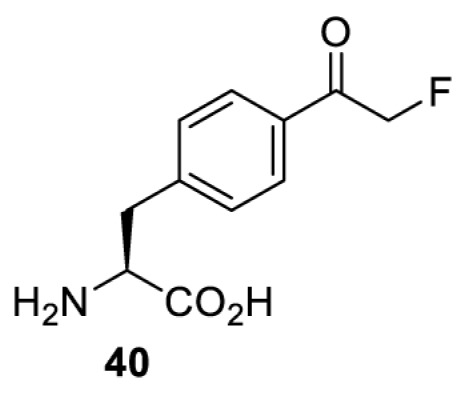	*Ec*TyrRS [84,101]	Y37I D182G F183M L186A D265R	${BstRNA}_{CUA}^{Tyr}$	Chemical crosslinking [84,101] Method development [101]
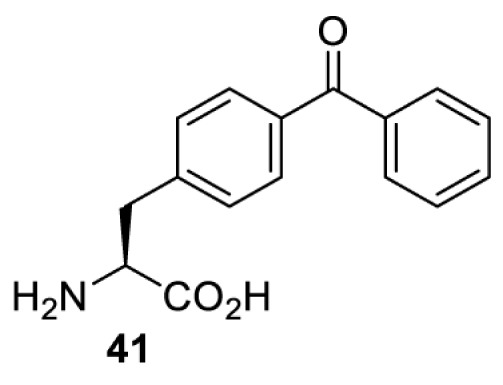	*Ec*TyrRS [15,37,55,78,85,92,94,95,99,102–105]	Y37G D182G L186A D265R [78,85,103] Y37G D182G L186A [15,55,92,94,95,99,102,104,105] Y37G D182G F183Y L186M [37]	${EctRNA}_{CUA}^{Tyr}$ [15,78,103]	Mechanistic studies [15] Method development [37,78,95,99,103,106] Photocrosslinking [85,92,94,102,104,105] Photoinhibition [55]
			${BstRNA}_{CUA}^{Tyr}$ [37,55,85,92,94,95,99,102,104,105]	
	*Mm*PylRS [106]	A302T N346T C348T W417C [106]	${MmtRNA}_{CUA}^{Pyl}$ [106]	
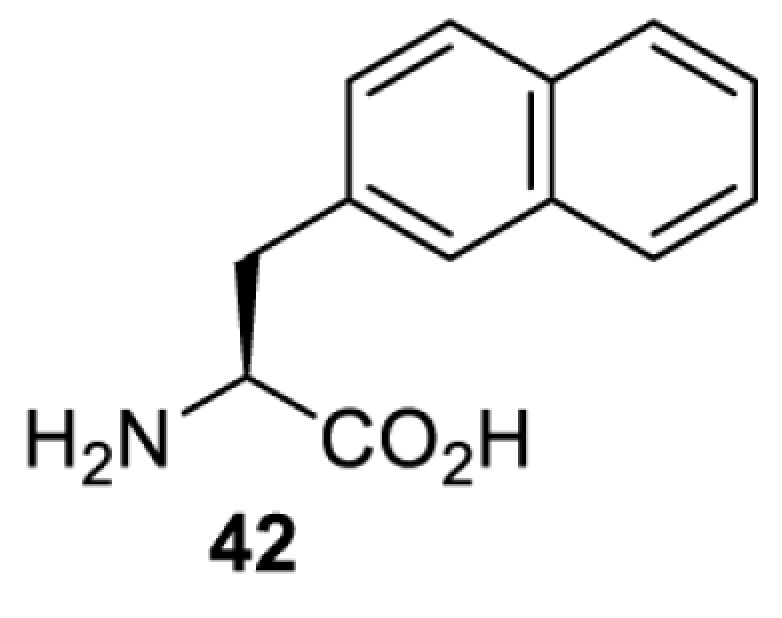	*Mm*PylRS [106]	A302T N346G C348T V401I W417Y	${MmtRNA}_{CUA}^{Pyl}$	Method development
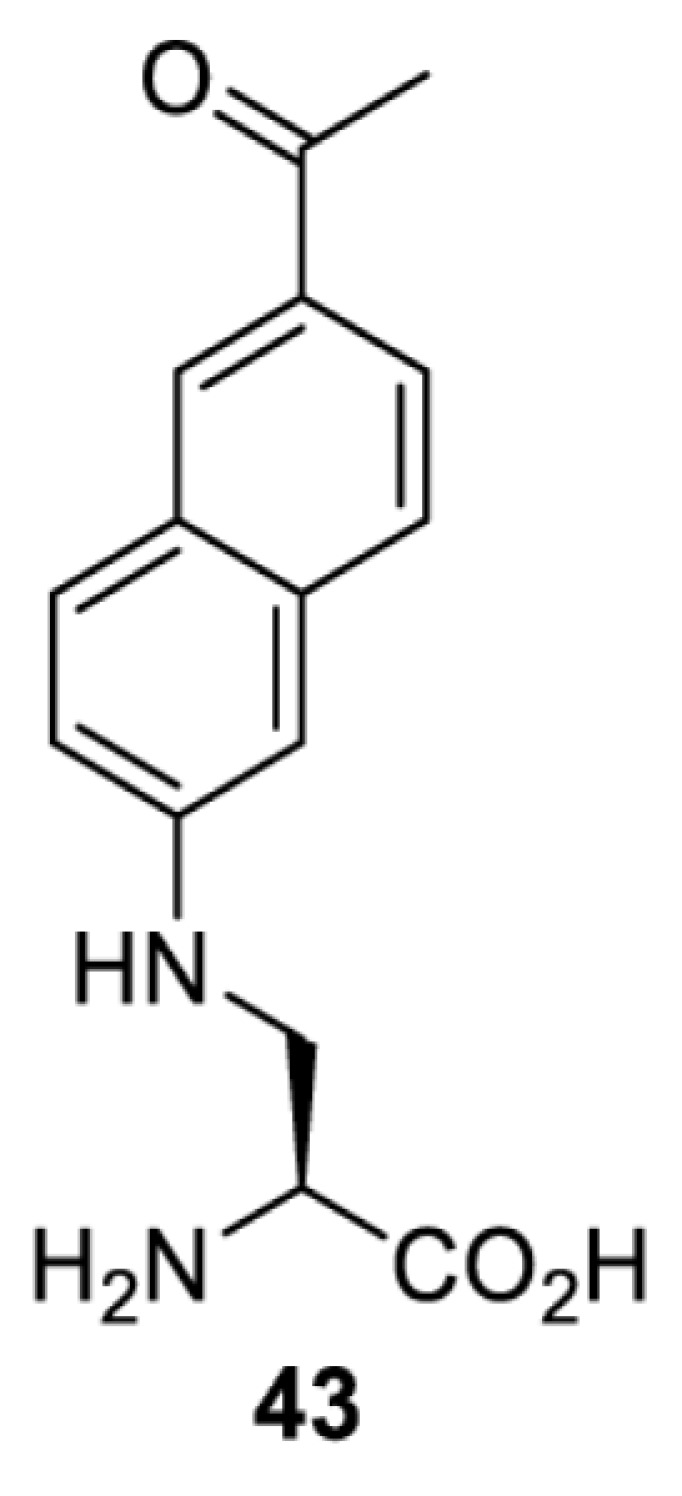	*Ec*LeuRS [107–109]	L38F M40G L41P Y499V Y500L Y527A H537E L538S F541C A560V	${EctRNA}_{CUA}^{Leu}$	Method development [107,108] Spectroscopic probe [109]
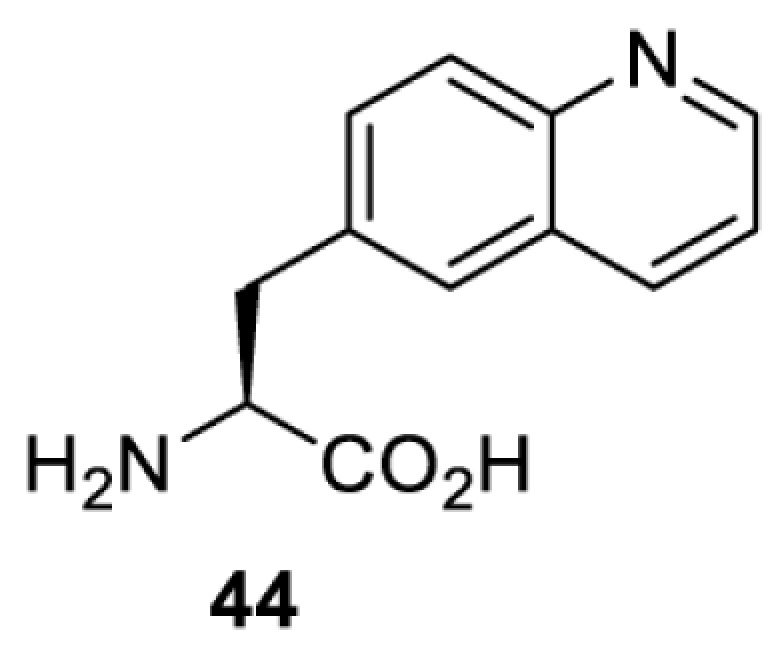	*Mm*PylRS [110]	N346Q C348S V401G W417T	${MmtRNA}_{CUA}^{Pyl}$	Spectroscopic Probe
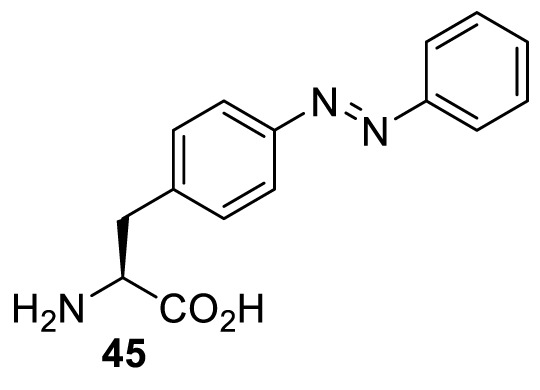	*Mb*PylRS [111]	L270F L274M N311G C313G Y349F	${MbtRNA}_{CUA}^{Pyl}$	Photoswitching
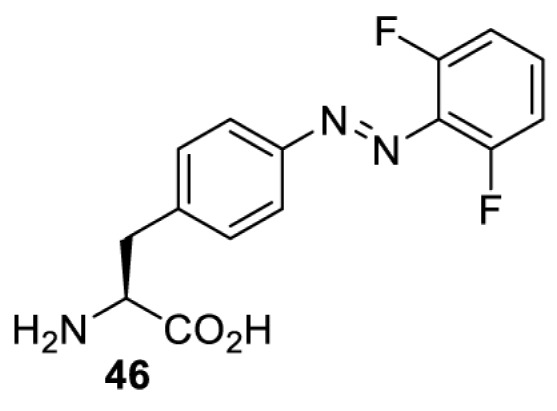	*Mb*PylRS [111]	L270F L274M N311G C313G Y349F	${MbtRNA}_{CUA}^{Pyl}$	Photoswitching
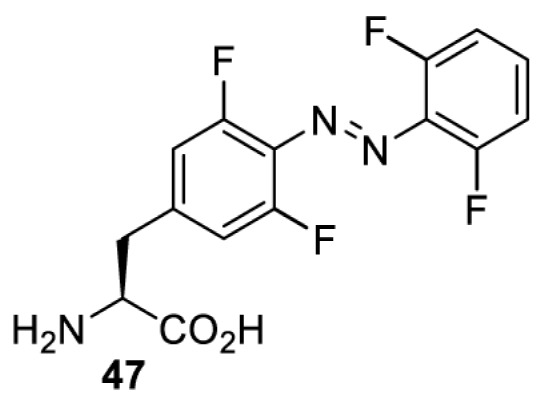	*Mb*PylRS [111]	L270F L274M N311G C313G Y349F	${MbtRNA}_{CUA}^{Pyl}$	Photoswitching
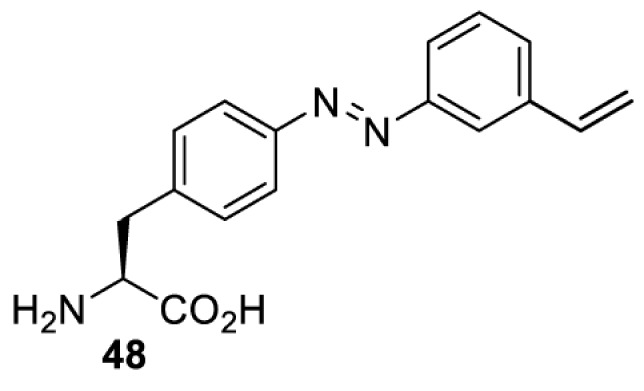	*Mm*PylRS [56,112]	A302T L309S N346V C348G	${MmtRNA}_{CUA}^{Pyl}$	Method development [112] Photoswitching [56]
**Histidine derivatives**				
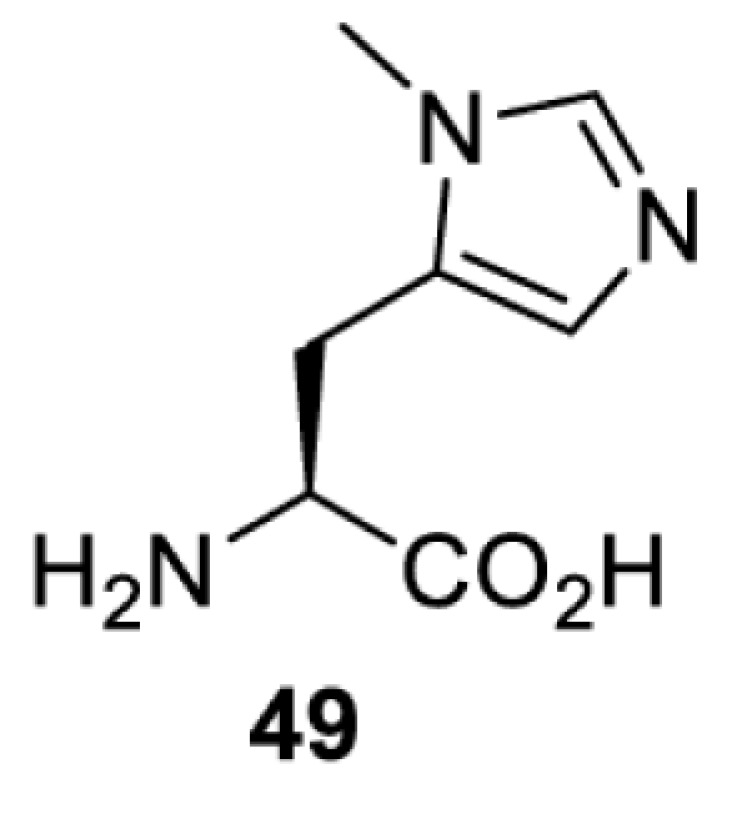	*Ma*PylRS [26]	L121M L125I Y126F M129A V168F	${MatRNA}_{CUA}^{Pyl}$	Method development
	*Mb*PylRS [113]	L270I Y271F L274G C313F Y349F	${MbtRNA}_{CUA}^{Pyl}$	
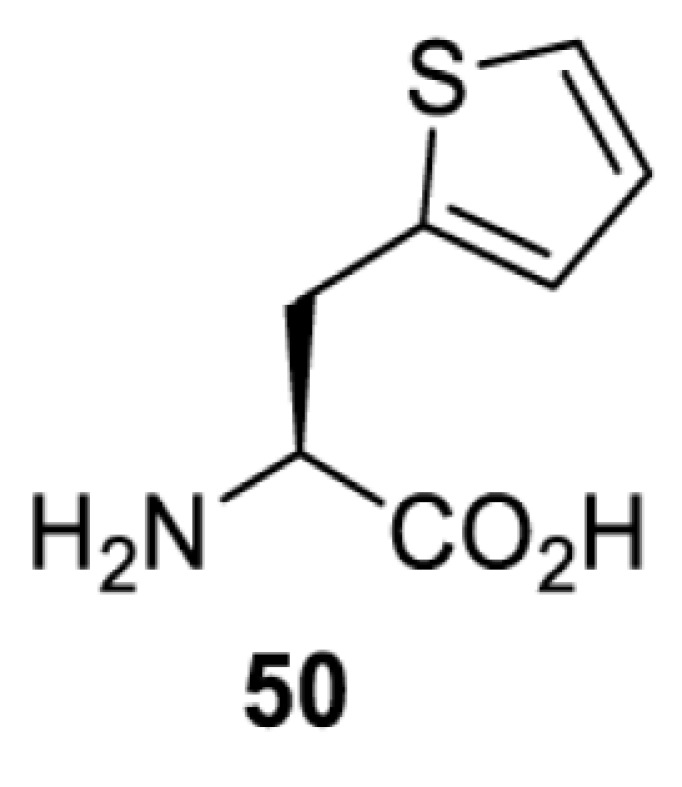	*Mb*PylRS [113]	L270I Y271F L274G C313F Y349F	${MbtRNA}_{CUA}^{Pyl}$	Method development
**Lysine derivatives**				
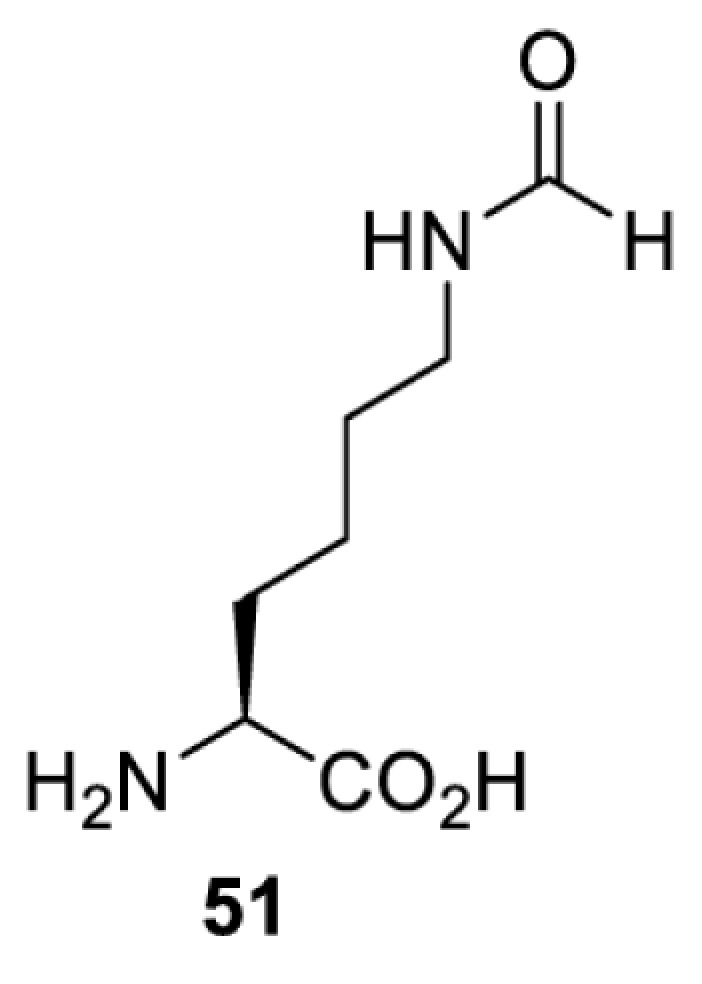	*Mb*PylRS [114]	L266M L270I Y271F L274A C313F	${MbtRNA}_{CUA}^{Pyl}$	Method development
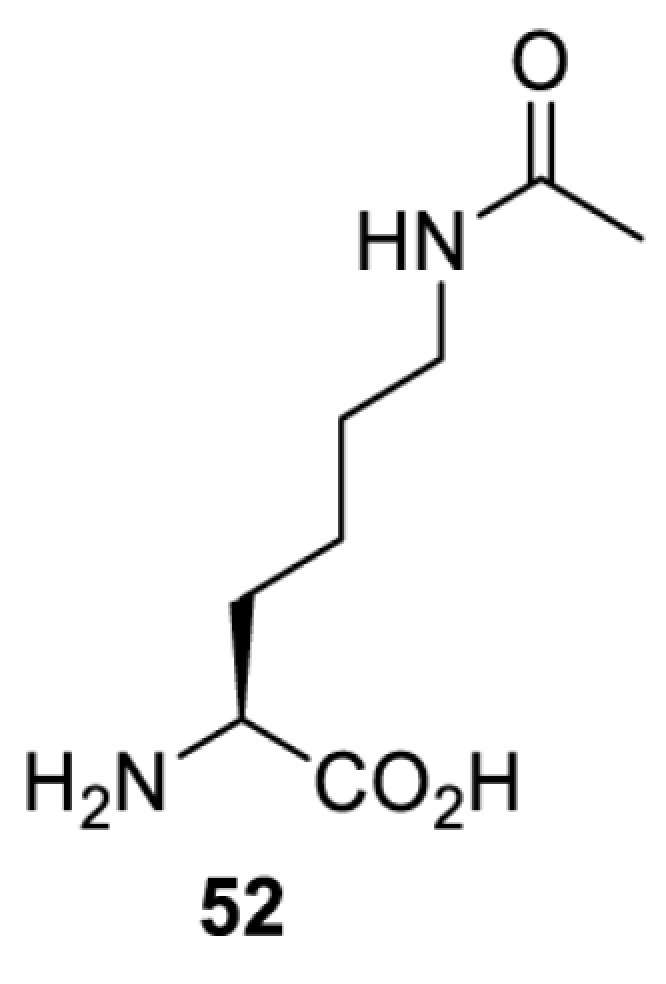	*Mb*PylRS [115–117]	D76G L266V L270I Y271F L274A C313F [115] D76G L266M L270I Y271F L274A C313F [116,117]	${MbtRNA}_{CUA}^{Pyl}$	Method development [44,50,115,117,118] Spectroscopic probe [116]
	*Mm*PylRS [44,50,118]	L305I Y306F L309A C348F [118] L301M Y306L L309A C348F [44,50]	${MmtRNA}_{CUA}^{Pyl}$ [50,118] ${MmtRNA}_{UUA}^{Pyl}$ [44]	
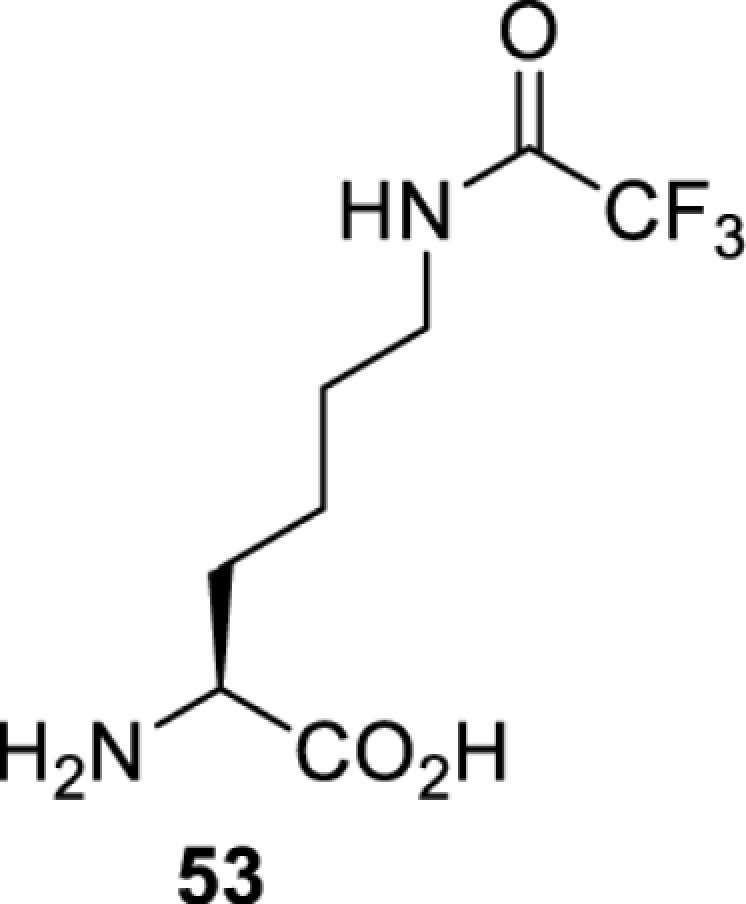	*Mm*PylRS [50]	L301M Y306L L309A C348F	${MmtRNA}_{CUA}^{Pyl}$	Method development
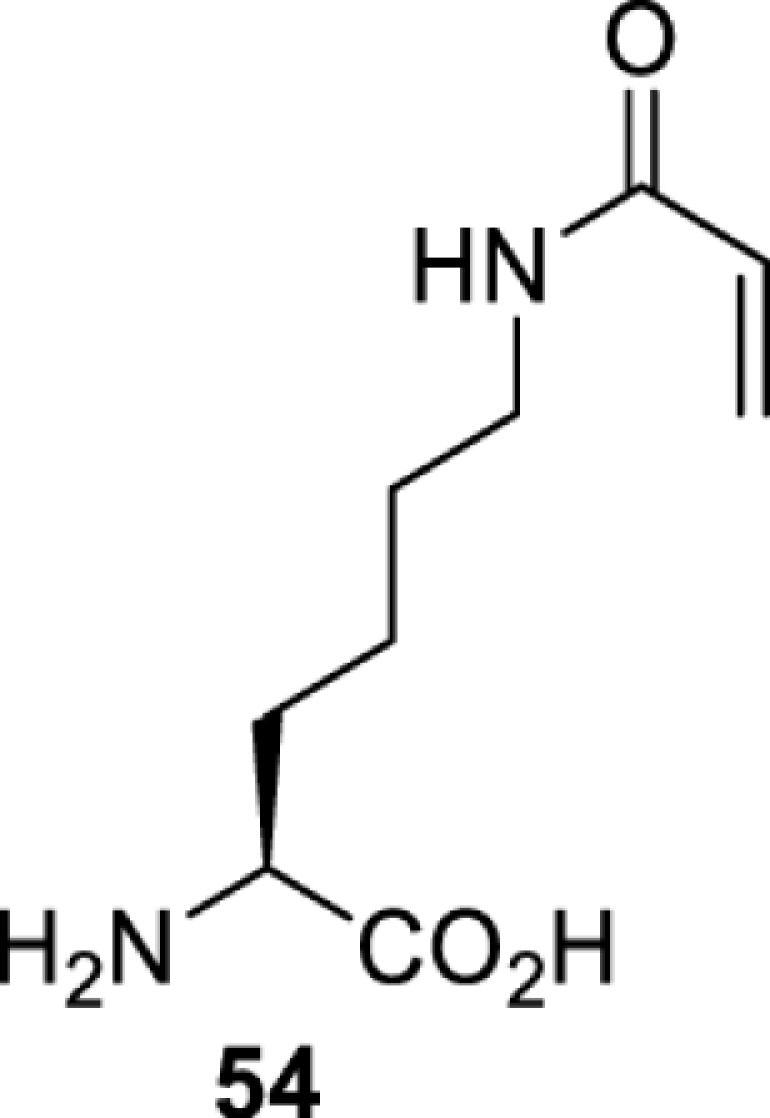	*Mb*PylRS [119]	D76G L266M L270I Y271F L274A C313F	${MbtRNA}_{CUA}^{Pyl}$	Method development
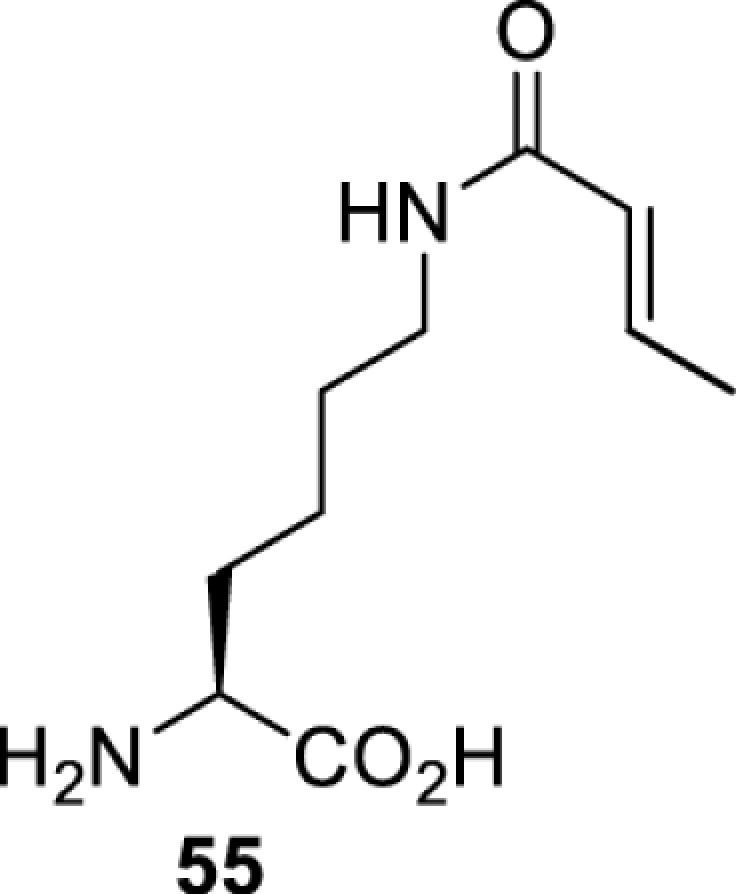	*Mb*PylRS [77,120]	L274A C313F Y349F [120] wt [77]	${MbtRNA}_{CUA}^{Pyl}$	Method development
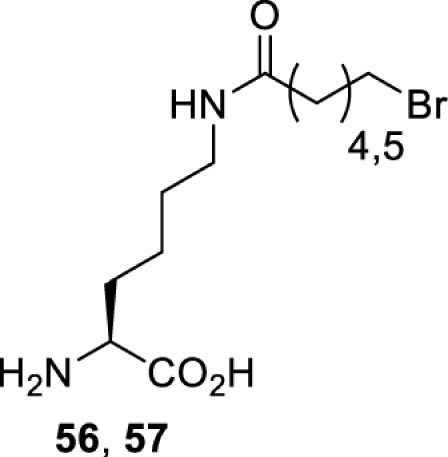	*Mb*PylRS [121]	Y271M L274A C313A	${MbtRNA}_{CUA}^{Pyl}$	Photocrosslinking
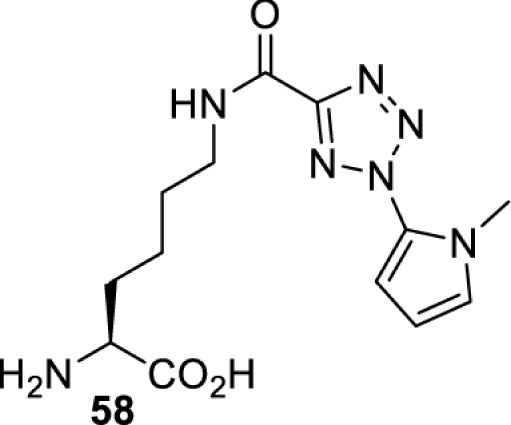	*Mm*PylRS [122]	Y306V L309A C348F Y384F	${MmtRNA}_{CUA}^{Pyl}$	Photocrosslinking
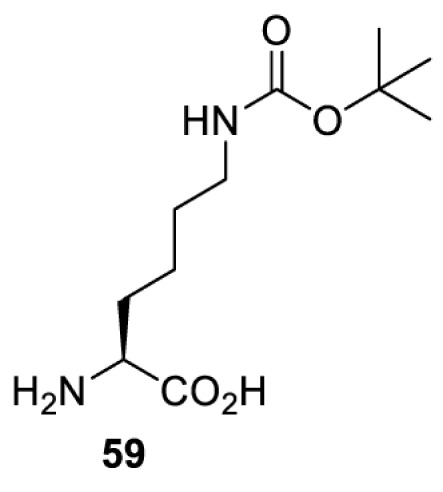	*Ma*PylRS [26]	wt	${MatRNA}_{CUA}^{Pyl}$	Method development [21,22,25,26,44,47,69,73,77,106,115,117,118,123–130]
	*Mb*PylRS [21,25,44,125,129,130]	wt	${MbtRNA}_{CUA}^{Pyl}$ [25,44,125,129,130] ${MbtRNA}_{UUA}^{Pyl}$ [44] ${MbtRNA}_{UCA}^{Pyl}$ [25,44]	
	*Mm*PylRS [22,26,44,47,69,73,77,106,115,117,118,123,124,126–128]	wt	${MmtRNA}_{CUA}^{Pyl}$ [21,22,26,47,69,73,77,106,115,118,123,124,126,127] ${MmtRNA}_{CUA}^{Pyl}BU25CB$[22,117,128] ${MmtRNA}_{UUA}^{Pyl}$ [21,44] ${MmtRNA}_{UCA}^{Pyl}$ [21]	
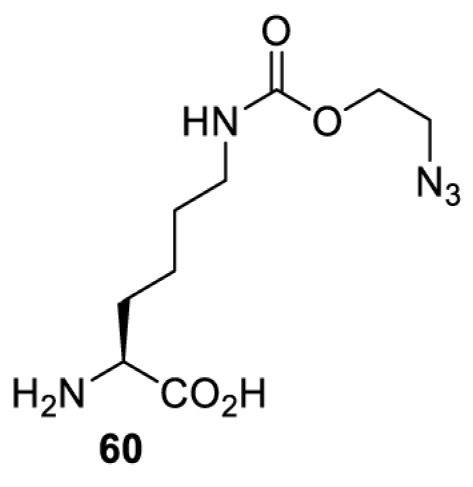	*Mb*PylRS [21,24,25,48,77,131–137]	Wt [21,24,25,48,77,132–136] L274A C313S Y349F [131] Y349F [137]	${MbtRNA}_{CUA}^{Pyl}$ [25,77,131] ${MbtRNA}_{UCA}^{Pyl}$ [24,25,48,132–136]	Bioorthogonal labelling [127,131,137] Imaging [136] Method development [24,25,77,134,137] Protein engineering [21,48,132,133,135–137]
	*Mm*PylRS [127]	wt	${MmtRNA}_{CUA}^{Pyl}$ [127,137] ${MmtRNA}_{UUA}^{Pyl}$ [21]	
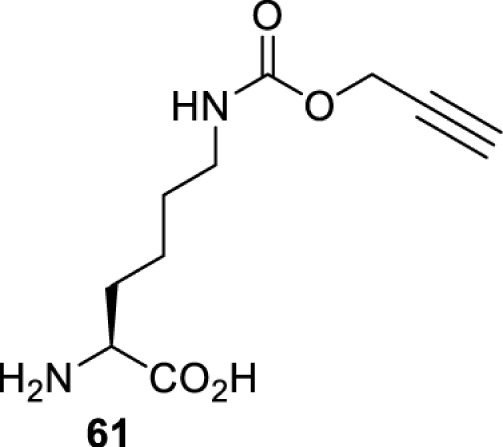	*Mb*PylRS [57,69,77,138,139]	wt	${MbtRNA}_{CUA}^{Pyl}$	Bioorthogonal labelling [131,139] Chemical decaging [57] Imaging [69,129,138] Method development [69,77]
	*Mm*PylRS [69,129,131]	wt	${MmtRNA}_{CUA}^{Pyl}$	
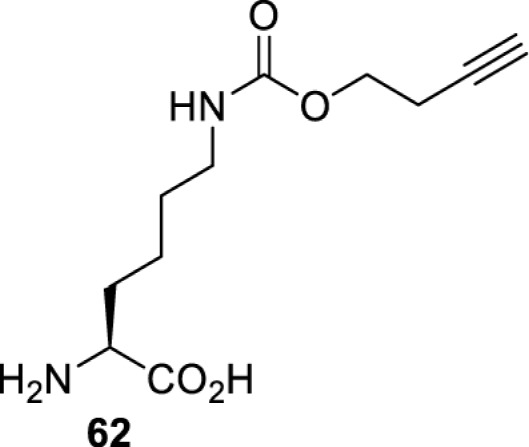	*Mb*PylRS [25,130]	wt	${MbtRNA}_{CUA}^{Pyl}$ [25]	Bioorthogonal labelling [130] Method development [25]
	*Mm*PylRS [130]	wt	${MmtRNA}_{CUA}^{Pyl}$	
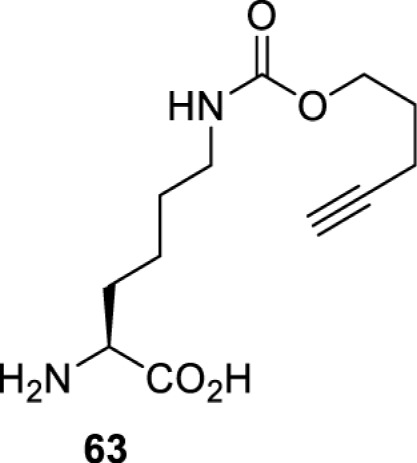	*Mb*PylRS [131,140]	L274A C313S Y349F	${MbtRNA}_{CUA}^{Pyl}$	Bioorthogonal labelling
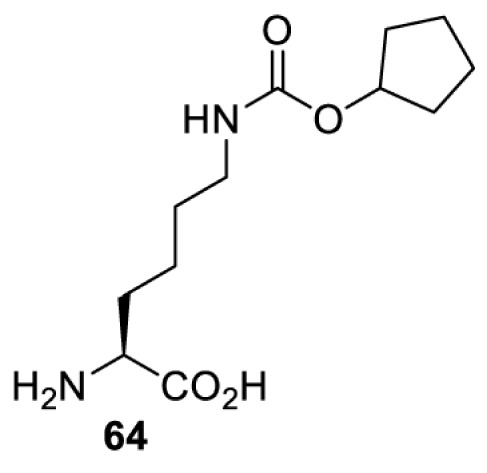	*Mm*PylRS [141]	wt	${MmtRNA}_{CUA}^{Pyl}$	Method development
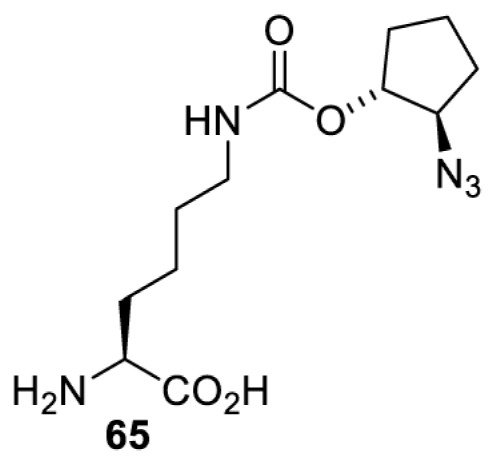	*Mb*PylRS [140]	wt	${MbtRNA}_{CUA}^{Pyl}$	Method development
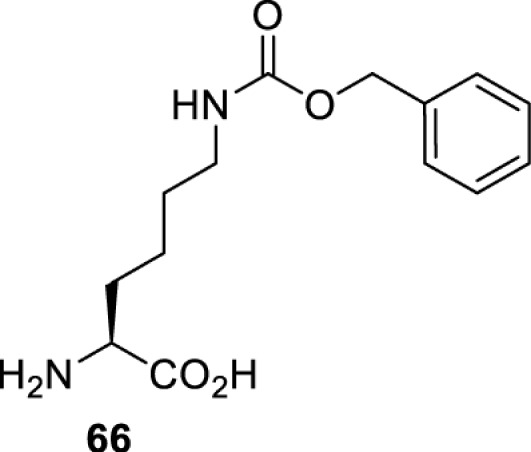	*Mm*PylRS [118,142,143]	R61K G131E L309A C348V Y384F [118] Y306A Y384F [142] R61K G131E Y306A Y384F [143]	${MmtRNA}_{CUA}^{Pyl}$ [118,142] ${MmtRNA}_{CUA}^{Pyl}BU25C$ [143]	Method development [118,142,143]
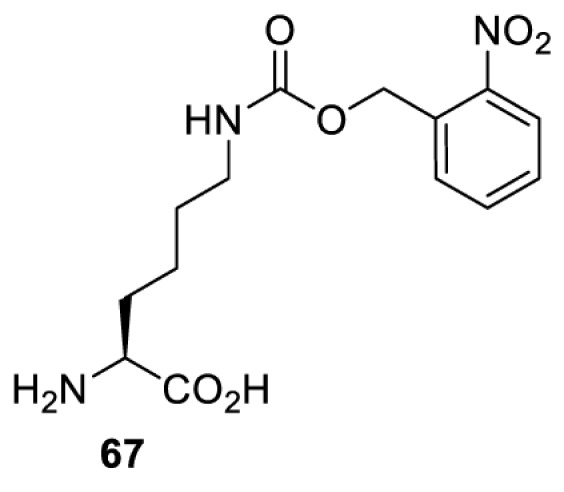	*Mb*PylRS [140]	Y271I L274A C313A Y349F	${MbtRNA}_{CUA}^{Pyl}$	Method development [140,141] Photoactivation [61,144]
	*Mm*PylRS [61,141,144]	Y306M L309A C348A Y384F	${MmtRNA}_{CUA}^{Pyl}$	
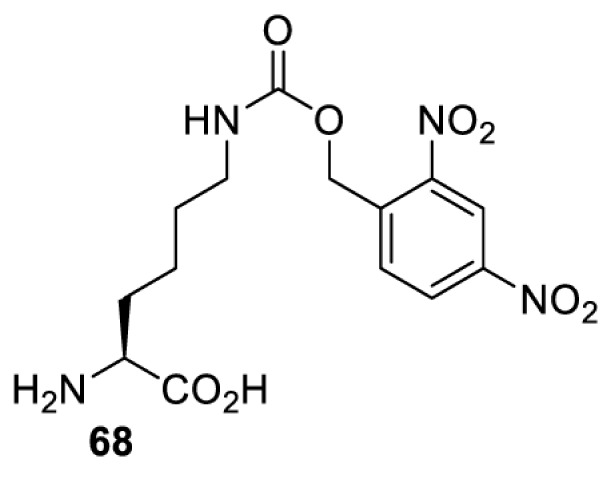	*Mb*PylRS [145]	Y271M L274T C313A Y349F	${MbtRNA}_{CUA}^{Pyl}$	Method development
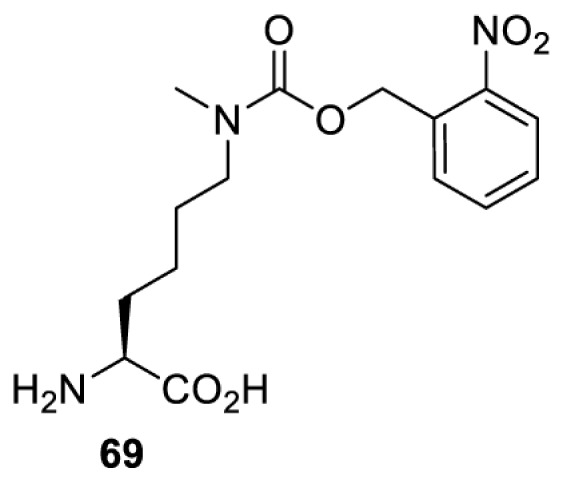	*Mb*PylRS [146]	Y271I 274M C313A	${MbtRNA}_{CUA}^{Pyl}$	Method development
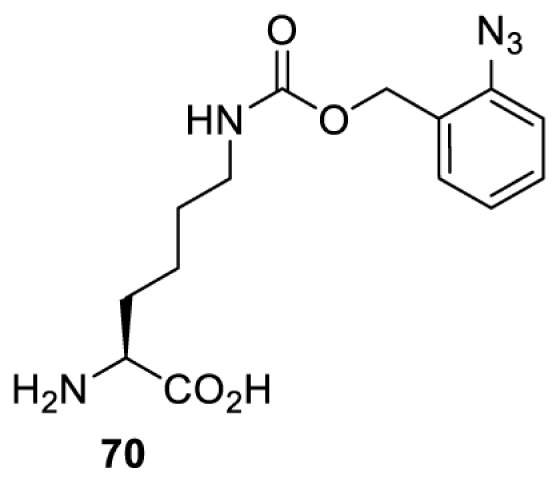	*Mb*PylRS [63]	Y271A Y349F	${MbtRNA}_{CUA}^{Pyl}$	Chemical decaging
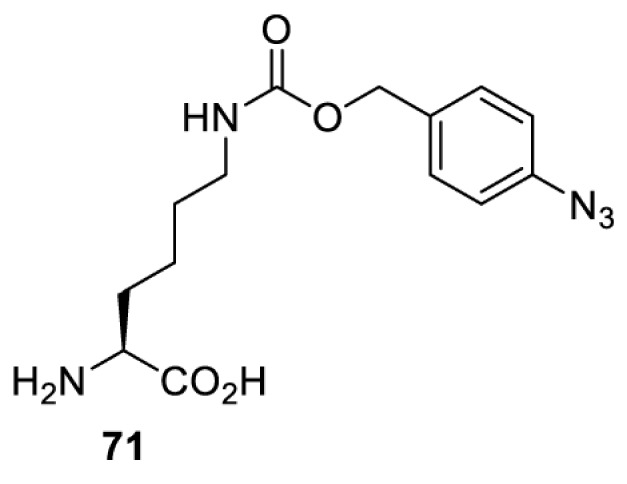	*Mb*PylRS [62]	L274A C313S Y349F	${MbtRNA}_{CUA}^{Pyl}$	Bioorthogonal labelling Chemical decaging
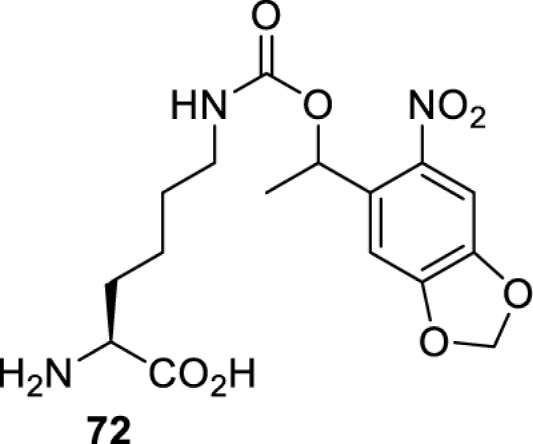	*Mb*PylRS [66,67,69,75,125,147–152]	M241F A267S Y271C L274M [66,67,69,75,125,147–152]	${MbtRNA}_{CUA}^{Pyl}$ [66,67,69,75,125,147–152] ${MbtRNA}_{CUA}^{Pyl}U25C$ [66]	Method development [69] Photoactivation [66,67,75,125,147–152]
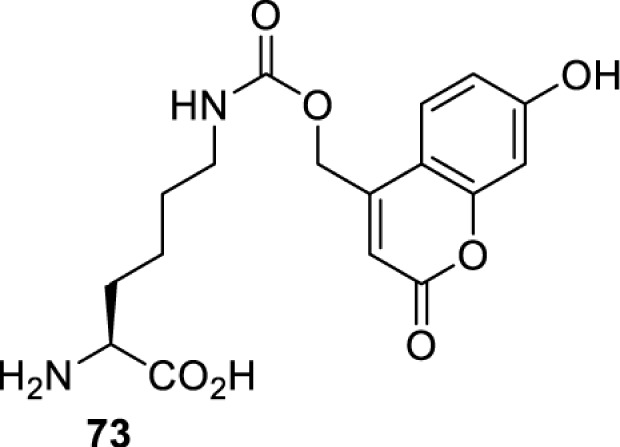	*Mb*PylRS [153]	Y271A L274M	${MbtRNA}_{CUA}^{Pyl}$	Photoactivation
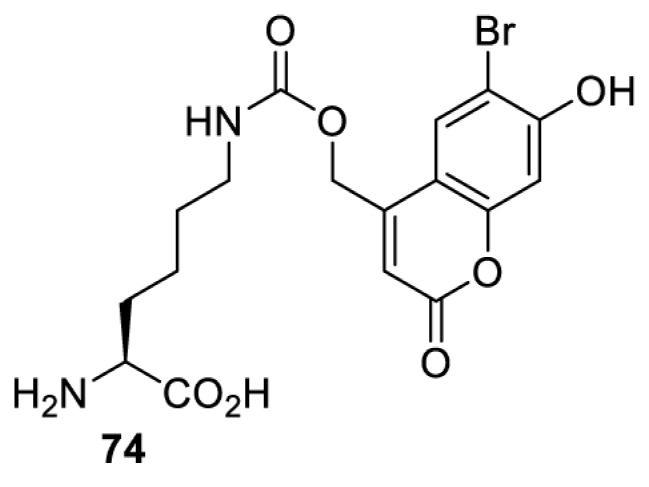	*Mb*PylRS [153]	Y271A L274M	${MbtRNA}_{CUA}^{Pyl}$	Photoactivation
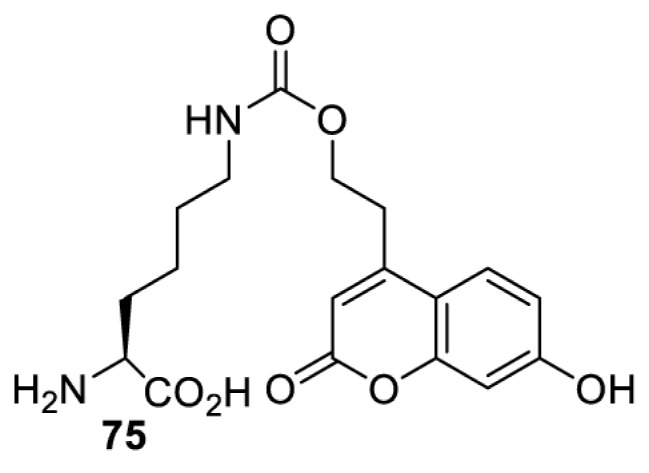	*Mb*PylRS [153]	Y271A L274M	${MbtRNA}_{CUA}^{Pyl}$	Method development
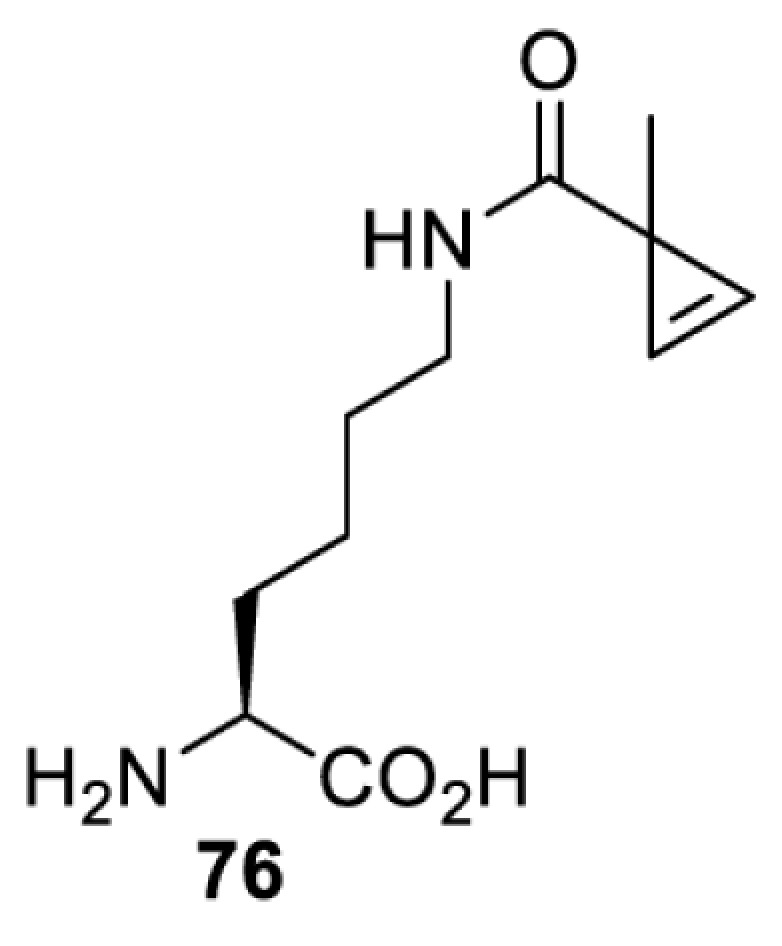	*Mb*PylRS [154]	L266M L270I Y271L L274A C313	${MbtRNA}_{CUA}^{Pyl}$	Method development
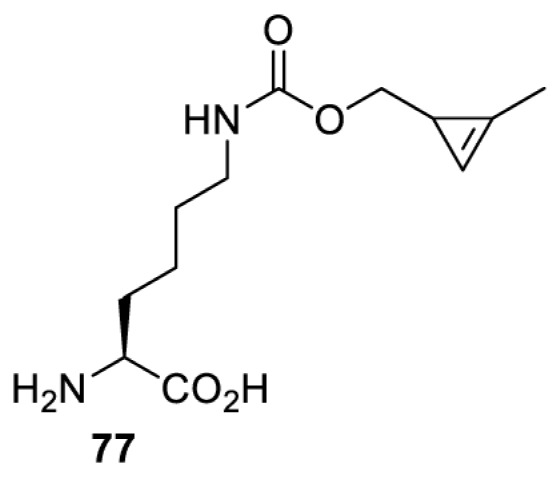	*Mb*PylRS [24,25]	wt	${MbtRNA}_{CUA}^{Pyl}$ [25] ${MbtRNA}_{UCA}^{Pyl}$ [24]	Imaging [123] Method development [22,24,25,69,115,155,156]
	*Mm*PylRS [22,69,115,123,155,156]	Wt [22,69,115,123,155,156] Y306A Y384F [155]	${MmtRNA}_{CUA}^{Pyl}BU25CB$[22,155] ${MmtRNA}_{CUA}^{Pyl}$ [69,115,123,156]	
	*Mx1201*PylRS [155]	wt	${Mx1201tRNA}_{CUA}^{Pyl}$ ${Mx1201tRNA}_{CUA}^{Pyl}$C41CA	
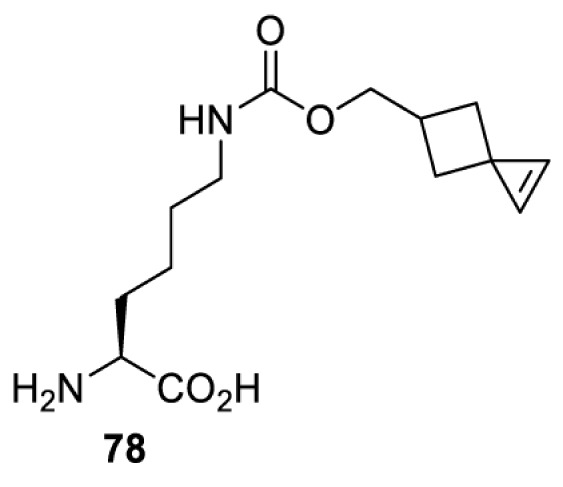	*Mm*PylRS [124]	wt	${MmtRNA}_{CUA}^{Pyl}$	Bioorthogonal labelling
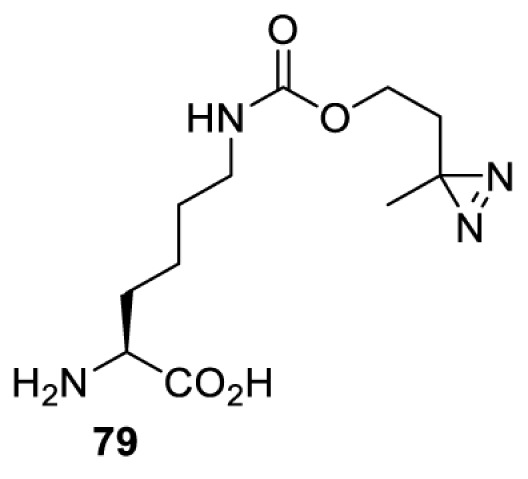	*Mb*PylRS [77,157,158]	Wt [77,158] L274M 313A Y349F [157]	${MbtRNA}_{CUA}^{Pyl}$	Method development [77,155,159] Photocrosslinking [157,158]
	*Mm*PylRS [155,159]	Y306A Y384F	${MmtRNA}_{CUA}^{Pyl}$ [159] ${MmtRNA}_{CUA}^{Pyl}$U25C [155]	
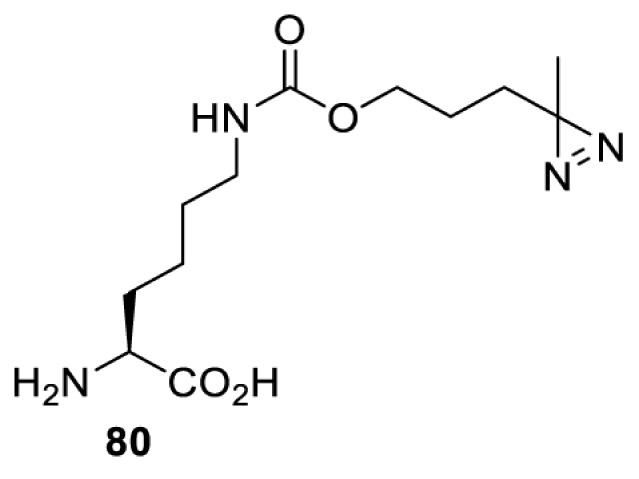	*Mm*PylRS [159]	Y306A Y384F	${MmtRNA}_{CUA}^{Pyl}$	Method development
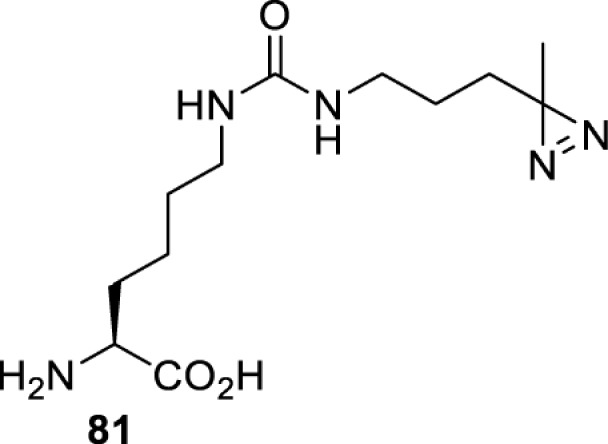	*Mb*PylRS [132,140,160]	L274A C313S Y349F	${MbtRNA}_{CUA}^{Pyl}$	Method development [140] Photocrosslinking [132,160] Protein engineering [132]
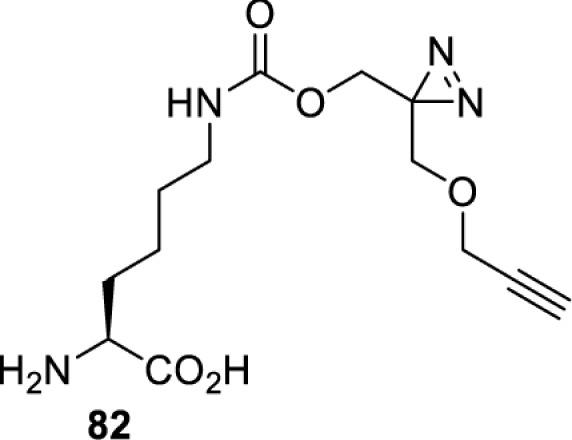	*Mm*PylRS [159]	Y306A Y384F	${MmtRNA}_{CUA}^{Pyl}$	Method development
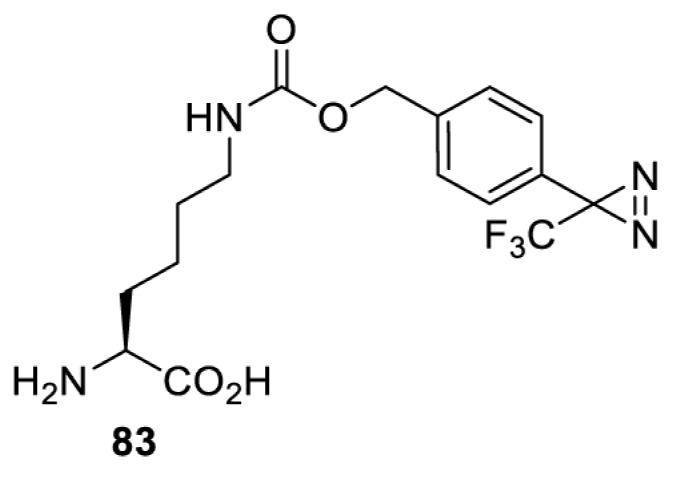	*Mm*PylRS [142]	Y306A Y384F	${MmtRNA}_{CUA}^{Pyl}$	Photocrosslinking
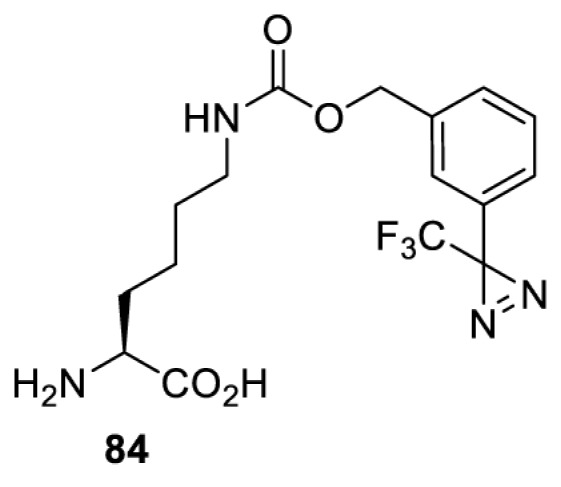	*Mm*PylRS [143]	R61K G131E Y306A Y384F	${MmtRNA}_{CUA}^{Pyl}BU25C$	Photocrosslinking
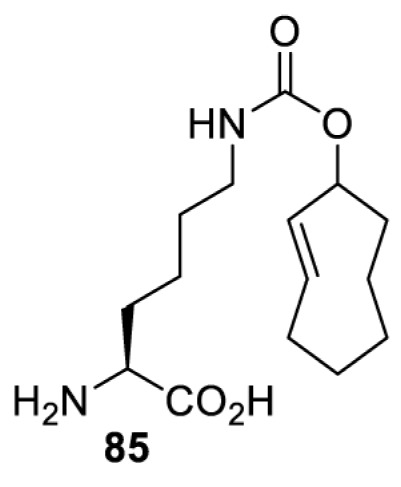	*Mm*PylRS [18,39,59–61,123,155,161–166]	Y306A Y384F [18,39,59–61,123,155,161–166]	${MmtRNA}_{CUA}^{Pyl}$ [18,39,59–61,123,161–166] ${MmtRNA}_{CUA}^{Pyl}BU25CB$[155]	Imaging [123,161,162,164,166] Chemical decaging [18,59–61] Chemical crosslinking [163] Method development [155,165] Protein labelling [39]
	*Mx1201*PylRS [155]	Y126A	${Mx1201tRNA}_{CUA}^{Pyl}$	
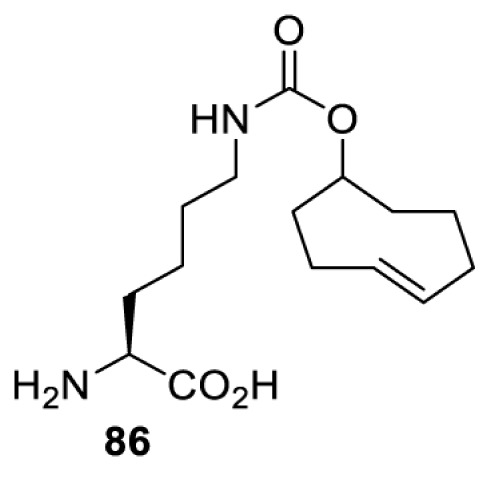	*Mb*PylRS [123,167,168]	Y271A L274M C313A	${MbtRNA}_{CUA}^{Pyl}$	Bioorthogonal labelling [124,167] Imaging [123,168] Method development [169]
	*Mm*PylRS [124,169]	Y306A Y384F [169] Y306A L309M C348A [124]	${MmtRNA}_{CUA}^{Pyl}$	
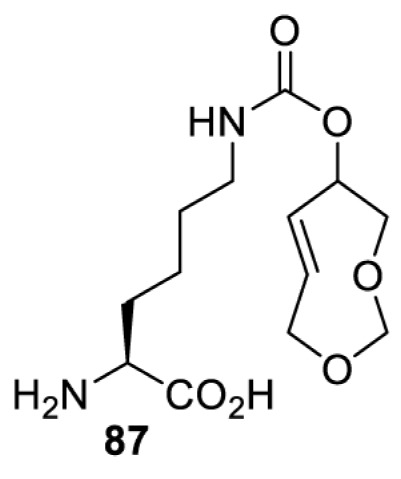	*Mm*PylRS [165]	Y306A Y384F	${MmtRNA}_{CUA}^{Pyl}$	Method development
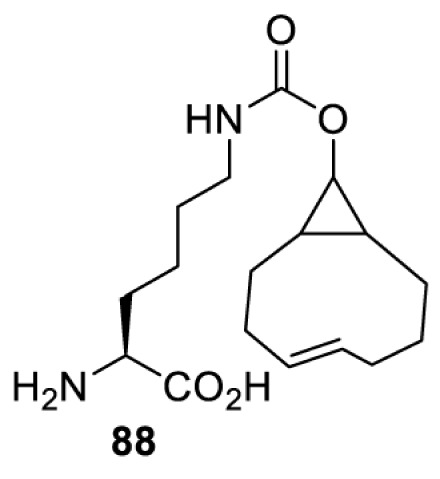	*Mb*PylRS [167,168]	Y271A L274M C313A	${MbtRNA}_{CUA}^{Pyl}$	Bioorthogonal labelling [167] Method development [167,169]
	*Mm*PylRS [169]	Y306A Y384F	${MmtRNA}_{CUA}^{Pyl}$	
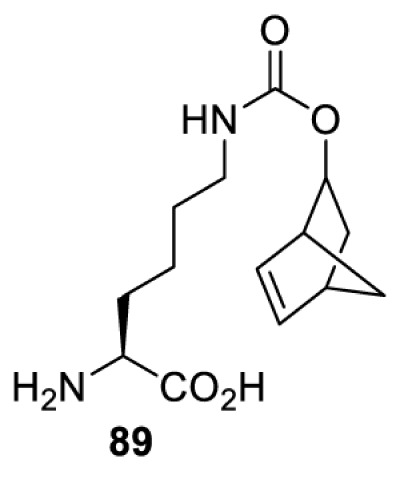	*Mb*PylRS [24]	wt	${MbtRNA}_{UCA}^{Pyl}$	Bioorthogonal labelling [39,127,131] Method development [24,169]
	*Mm*PylRS [39,127,131,169]	Wt [127,131] Y306A Y384F [39,169]	${MmtRNA}_{CUA}^{Pyl}$	
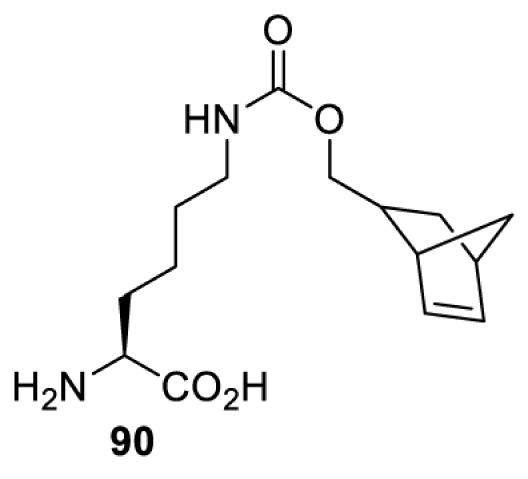	*Mm*PylRS [169]	Y306A Y384F	${MmtRNA}_{CUA}^{Pyl}$	Method development
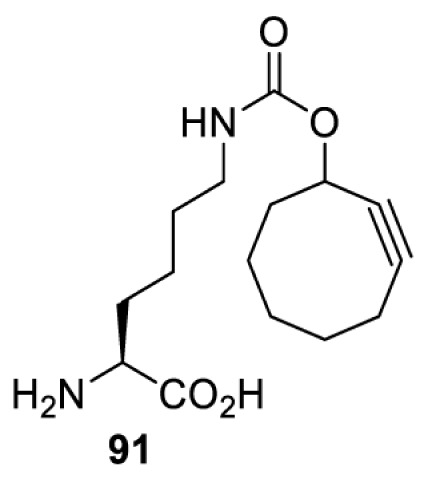	*Mm*PylRS [39,161,166,169,170]	Y306A Y384F	${MmtRNA}_{CUA}^{Pyl}$	Bioorthogonal labelling [39] Imaging [161,166,170] Method development [169]
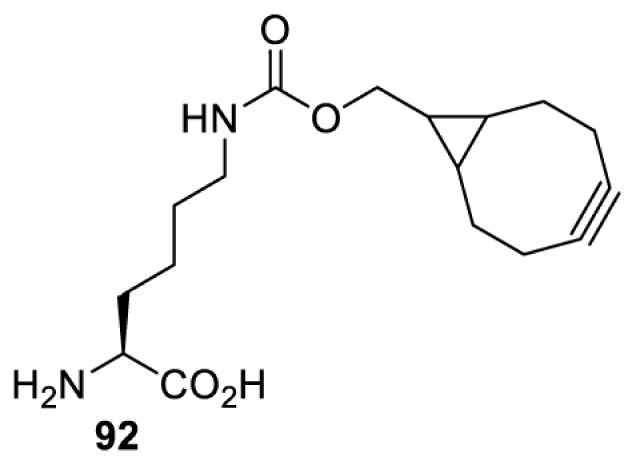	*Mb*PylRS [19,64,140,168,171,172]	Y271M L274G C313A [19,64,168,171,172] M241F A267S Y271C L274M [140]	${MbtRNA}_{CUA}^{Pyl}$ [64,140,168,171,172] ${MbtRNA}_{CUA}^{Pyl}U25C$ [19]	Chemical inhibition [64] Bioorthogonal labelling [39,131,167] Imaging [123,128,161,166,168,171,172] Method development [155,159,165] Protein engineering [140] Spectroscopic probe [19]
	*Mm*PylRS [39,123,128,131,155,159,161,165–167]	Y306A 384F [39,123,128,131,155,159,161, 165–167]	${MmtRNA}_{CUA}^{Pyl}$ [39,123,128,131,159,161, 165–167] ${MmtRNA}_{CUA}^{Pyl}BU25C$ [155]	
**Tryptophan derivatives**				
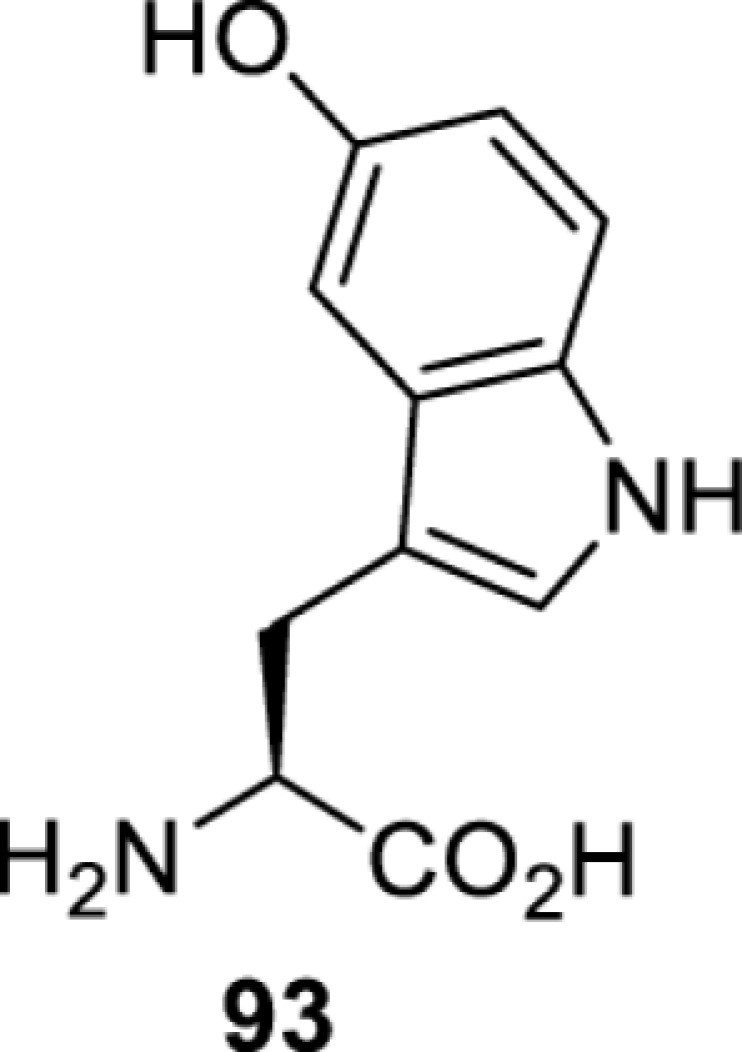	*Ec*TrpRS [34]	S8A V144S V146A S8A V144G V146C	${EctRNA}_{CUA}^{Trp}$	Method development
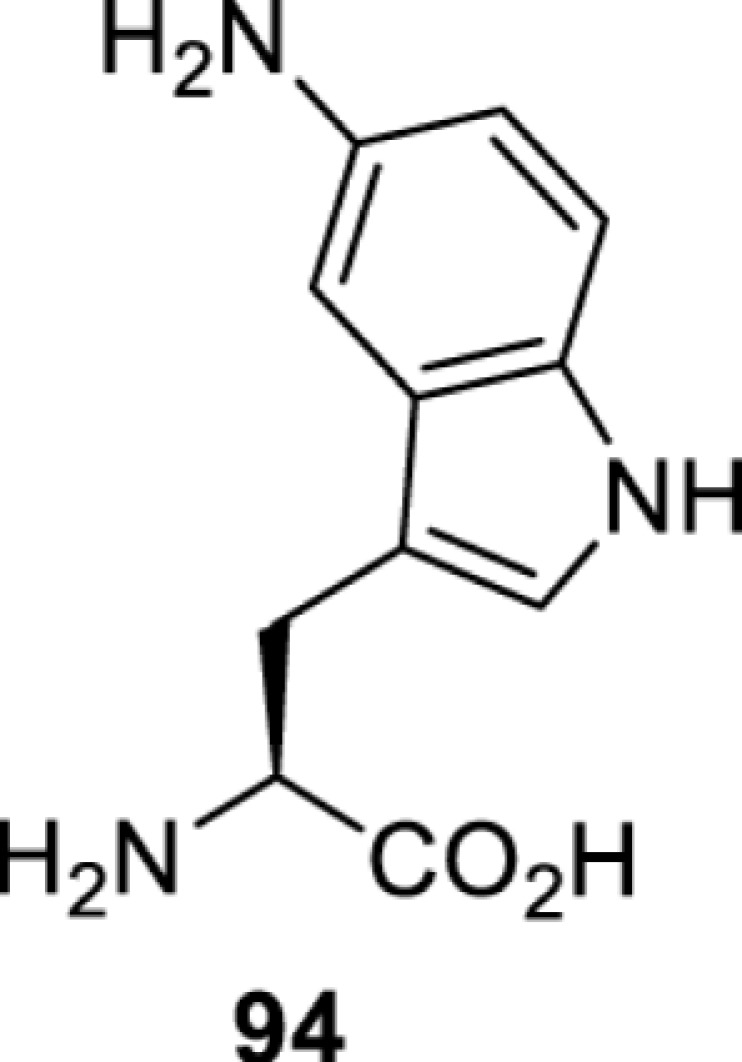	*Ec*TrpRS [34]	S8A V144S V146A	${EctRNA}_{CUA}^{Trp}$	Method development
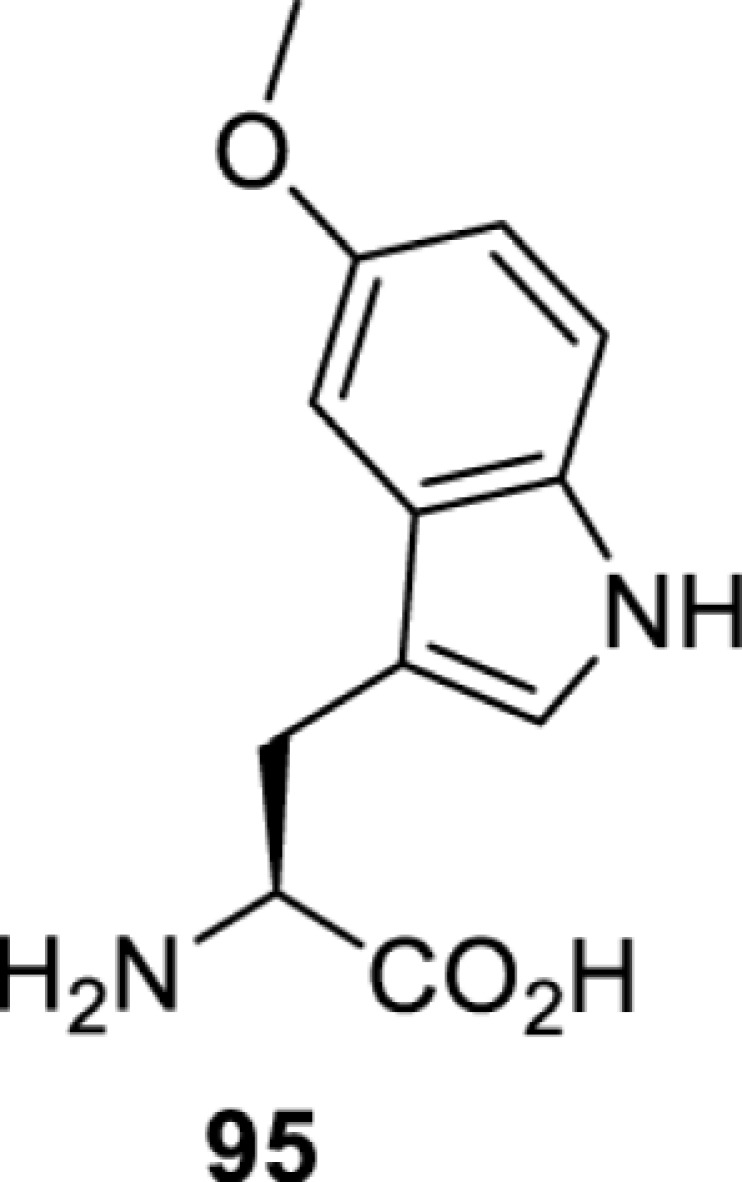	*Ec*TrpRS [34]	S8A V144G V146C	${EctRNA}_{CUA}^{Trp}$	Method development
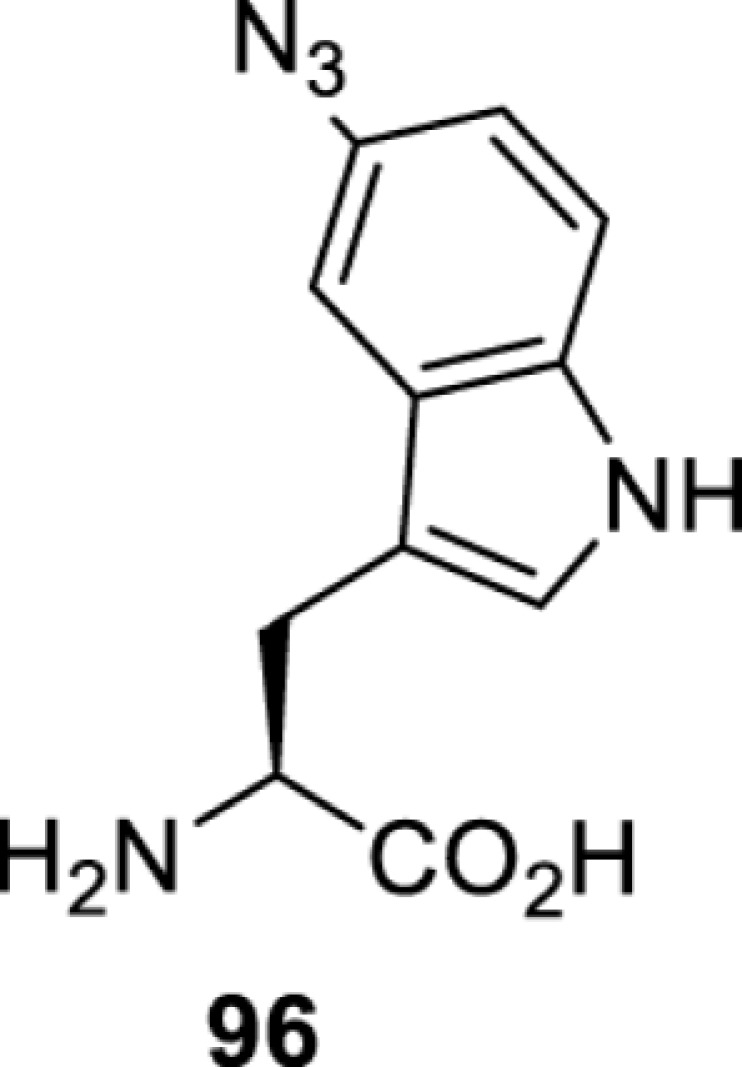	*Ec*TrpRS [34]	S8A V144G V146C	${EctRNA}_{CUA}^{Trp}$	Method development
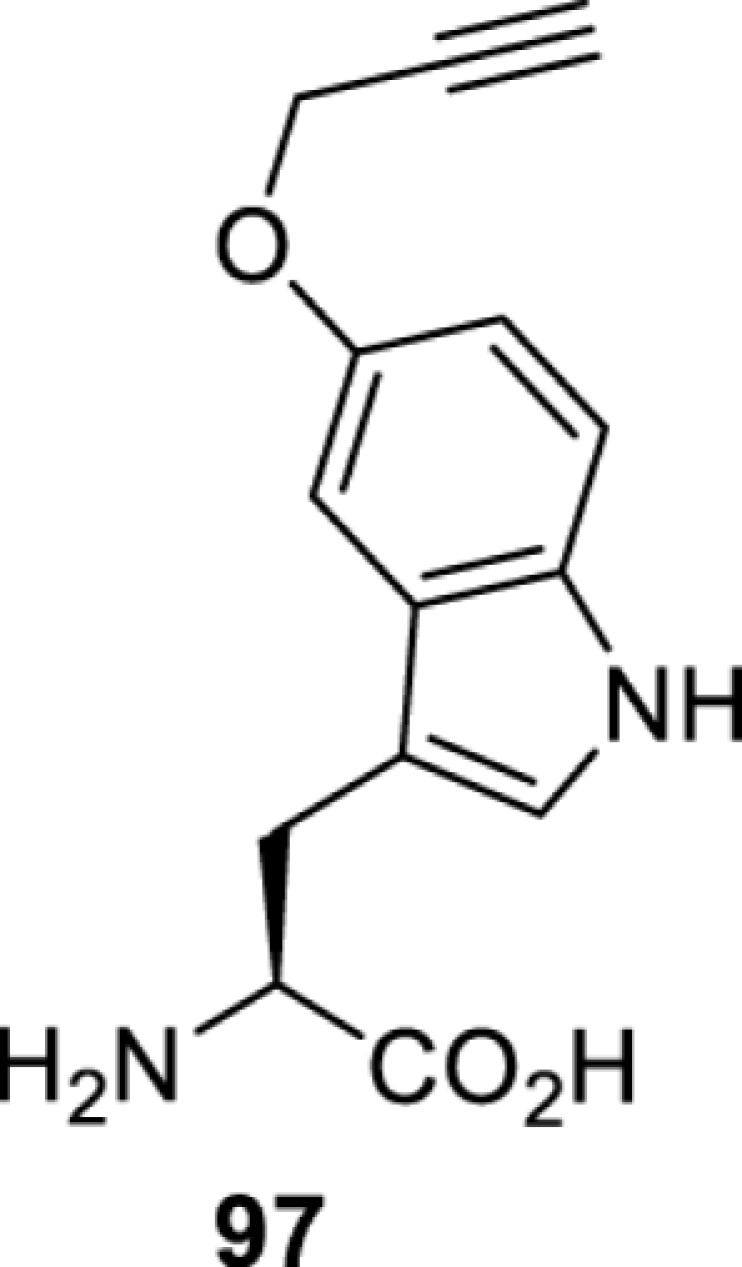	*Ec*TrpRS [34]	S8A V144G V146C	${EctRNA}_{CUA}^{Trp}$	Method development
**Tyrosine derivatives**				
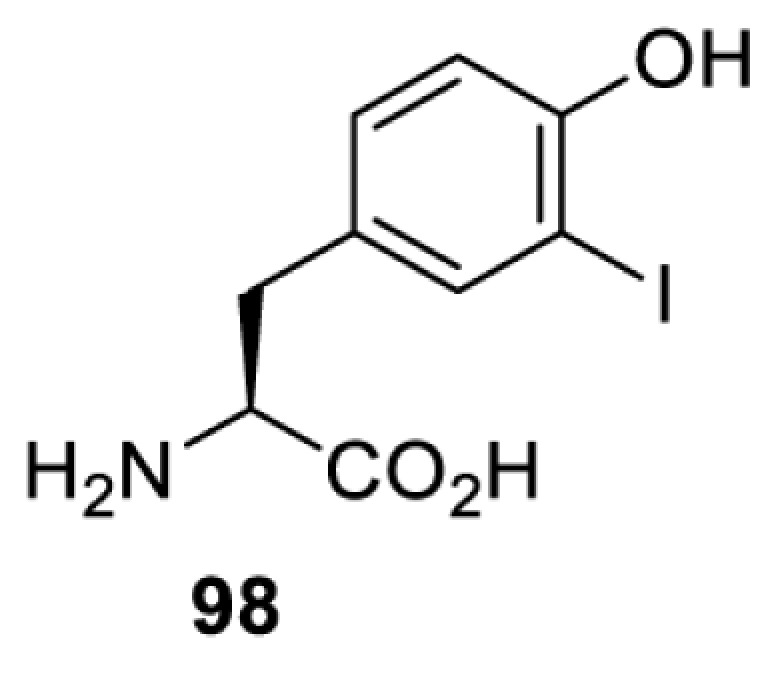	EcTyrRS [46]	Y37V Q195C	${BstRNA}_{CUA}^{Tyr}$	Method development
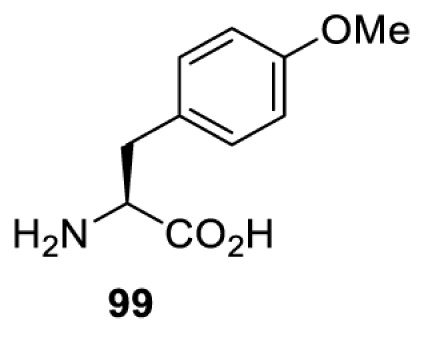	EcTyrRS_CUA_ [15,21,35,37,77,78,90]	Y37T D182T F183M D265R [78] Y37V D182S F183M [37] Y37V D182S F183M D265R [21,77,90] Y37T D182T F183M [15] Y37V D165G D182S F183M L186A D265R [35]	${EctRNA}_{CUA}^{Tyr}$ [15,21,35,77,78,90]	Mechanistic studies [15] Method development [21,25,35,37,77,78,90]
			${BstRNA}_{CUA}^{Tyr}$ [21,37,77,90]	
	*Ec*TyrRS_UCA_ [25]	Y37V D182S F183M	${EctRNA}_{UCA}^{Tyr}$	
			${BstRNA}_{UCA}^{Tyr}$	
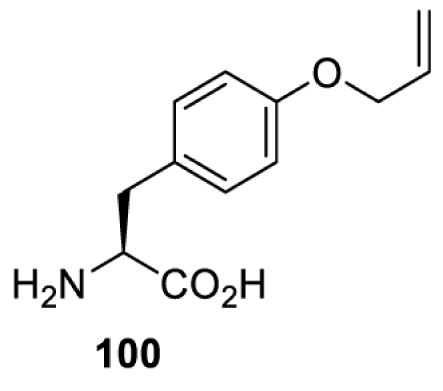	*Ec*TyrRS [77]	Y37V D182S F183M D265R	${EctRNA}_{CUA}^{Tyr}$	Method development
			${BstRNA}_{CUA}^{Tyr}$	
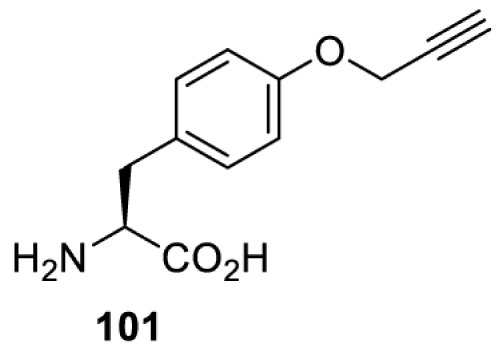	*Ec*TyrRS [25,35,37,77]	Y37V D182S F183M D265R [77] Y37S D182T F183M L186V [37] Y37V D182S F183M [25] Y37V D165G D182S F183M L186A D265R [35]	${EctRNA}_{CUA}^{Tyr}$ [25,35,77]	Method development
			${BstRNA}_{CUA}^{Tyr}$ [37,77]	
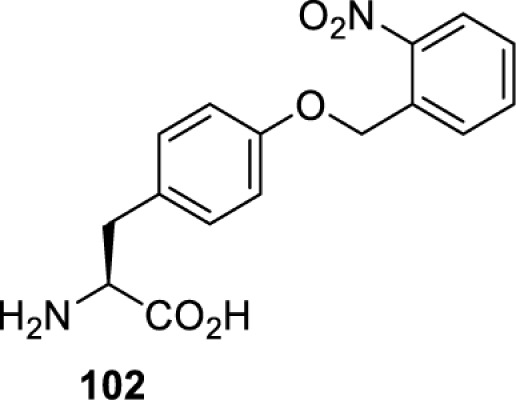	*Mb*PylRS [126,148,173]	L270F L274M N311G C313G Y349F [173] L270F L274M N311G C313G [126,148]	${MbtRNA}_{CUA}^{Pyl}$	Photoactivation
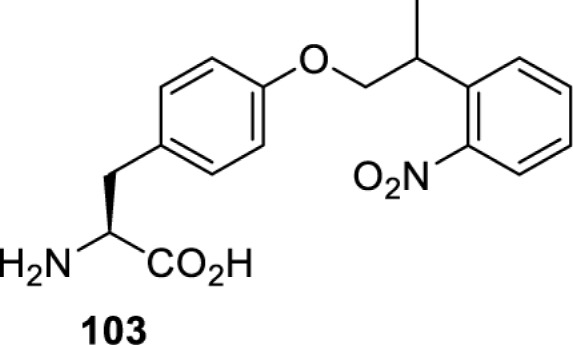	*Mb*PylRS [173]	L270F L274M N311G C313G Y349F	${MbtRNA}_{CUA}^{Pyl}$	Photoactivation
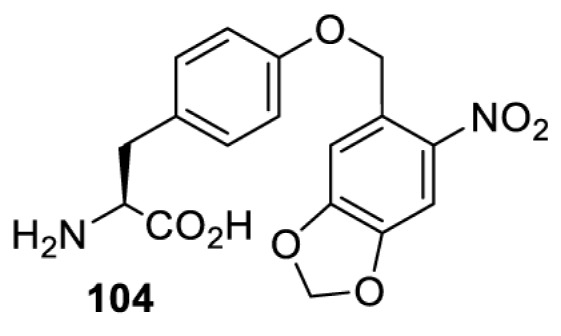	*Mb*PylRS [173]	L270F L274M N311G C313G Y349F	${MbtRNA}_{CUA}^{Pyl}$	Photoactivation
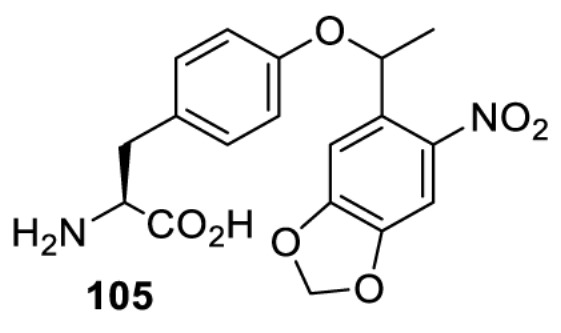	*Mb*PylRS [173]	L270F L274M N311G C313G Y349F	${MbtRNA}_{CUA}^{Pyl}$	Photoactivation
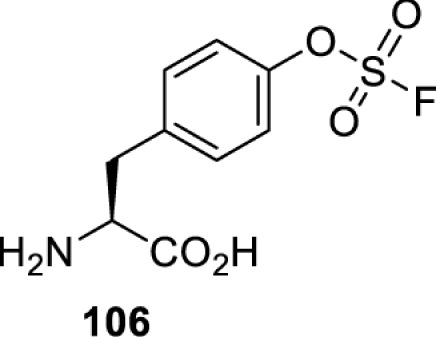	*Mm*PylRS [174]	N346T C348I Y384L W417K	${MmtRNA}_{CUA}^{Pyl}$	Bioorthogonal labelling
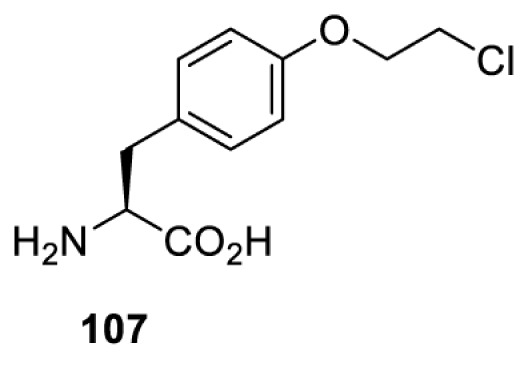	*Ec*TyrRS [35]	Y37V D182S F183M D265R Y37V D165G D182S F183M L186A D265R	${EctRNA}_{CUA}^{Tyr}$	Method development
**Miscellaneous unnatural amino acids**				
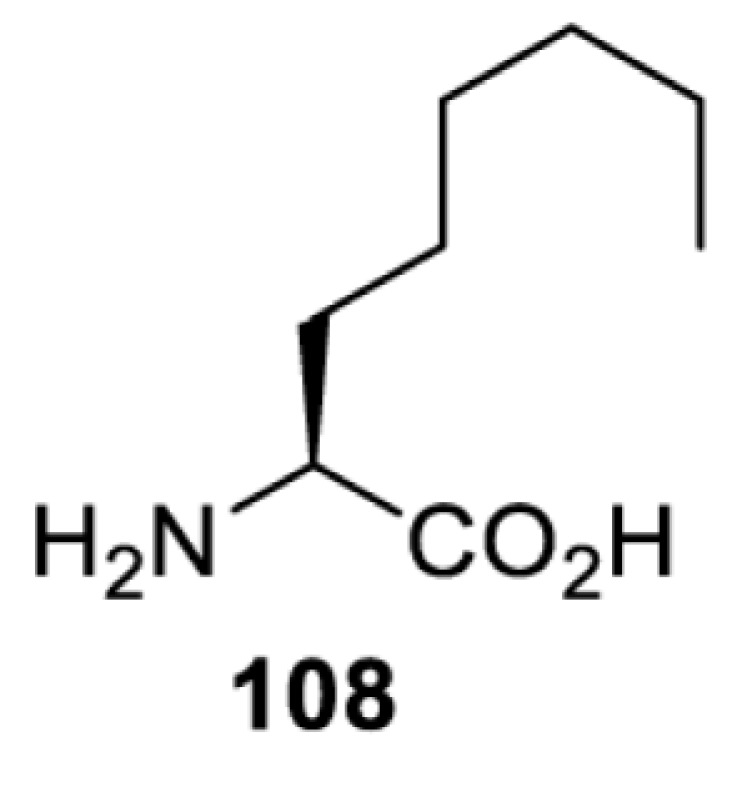	*Ec*LeuRS [24,25]	M40I T252A Y499I Y527A H529G [24] E20K M40V L41S T252R Y499S Y527L H529G H537G [25]	${EctRNA}_{CUA}^{Leu}$	Method development
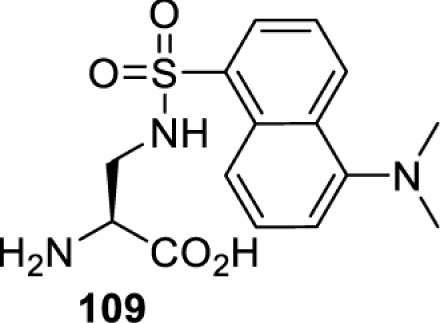	*Ec*LeuRS [15,103,175]	M40A L41N T252A Y499I Y527G H537T	${EctRNA}_{CUA}^{Leu}$	Mechanistic studies [15,103] Method development [175]
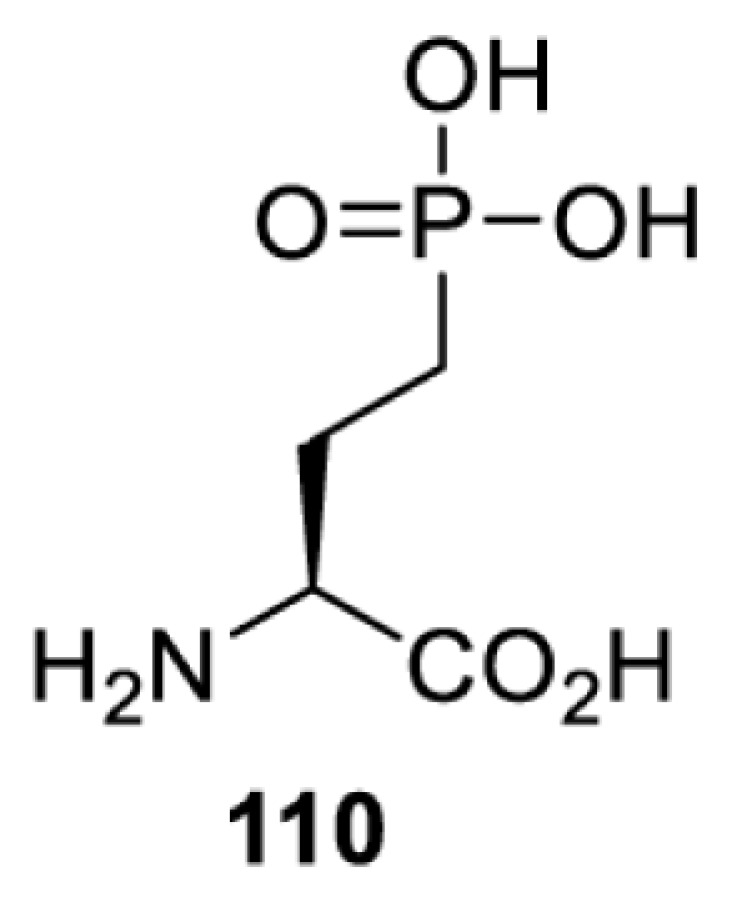	*Mm*SepRS [176]	wt	${MjtRNA}_{CUA}^{Cys}$	Method development

Method development includes demonstration of incorporation, optimisation of incorporation efficiency, application as a control substrate, proof-of-principle of a technique for subsequent studies etc. Abbreviations: Ma, *Methanomethylophilus alvus*; wt, wild type.

Many engineered *E. coli* aaRS/tRNA pairs have also been used as orthogonal pairs in mammalian cells. The most successful ones are the *E. coli* tyrosine, leucine and tryptophan pairs [33]. However, as all these synthetases naturally recognise a canonical amino acid, it is necessary to abolish their natural activity towards the canonical amino acid and to recognise only the designated unnatural amino acid. As it is technically difficult to perform directed evolution in mammalian cells due to low efficiency in transfection and screening, synthetase engineering is normally carried out in *E. coli* [34,35] or yeast [15,36,37] so that large mutant libraries can be easily screened. It is also necessary to modify the *E. coli* tRNA so that it decodes a blank codon instead of a codon corresponding to a canonical amino acid.

Based on the simplicity of the established methodology [38–40] and the promiscuity of many orthogonal synthetases towards different unnatural amino acids (*vide infra*) [41], the number of genetically incorporable unnatural amino acids has steadily increased. In addition, some orthogonal aaRS/tRNA pairs are mutually orthogonal [21,24–26,42] and can be used at the same time to incorporate multiple different unnatural amino acids into a protein of interest.

As recent reviews cover fundamental aspects of genetic code expansion [4,6,9,13], the engineering of new orthogonal synthetases [8], and general [5,10,11,14] or specific [7,12] applications of genetic code expansion in eukaryotic systems, we will focus on recent advances and applications of genetic code expansion for controlling protein function in mammalian cells through translational, optical or chemical means.

## Translational control by amber suppression

**Figure 3:**
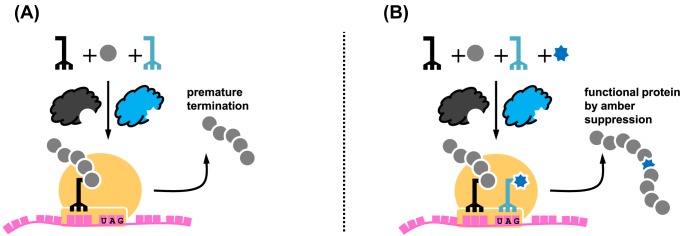
Use of amber suppression to switch on protein production (**A**) Absence of the unnatural amino acid leads to recognition of the UAG codon for translation termination. (**B**) Addition of the unnatural amino acid leads to amber suppression and successful production of the full-length and functional protein.

Genetic code expansion by unnatural amino acid incorporation in response to an amber stop codon provides the simplest way to ‘switch on’ protein production. In this case, an amber stop codon is placed into the gene of interest (Figure 3) for incorporation of the unnatural amino acid into a permissive site of the target protein [43]. In the absence of the designated unnatural amino acid, protein translation stops prematurely at the amber stop codon, generating truncated and non-functional protein product and thus giving an effect as a nonsense mutation (Figure 3A). In the presence of the unnatural amino acid, the orthogonal tRNA is acylated and decodes the amber codon, leading to amber suppression and generation of full-length, functional protein product (Figure 3B). Thus, simple addition of the unnatural amino acid into the growth medium ‘switches on’ the protein production and function [44]. In contrast with commonly used systems for inducible mammalian protein expression (e.g. the tetracycline transcriptional transactivation) [45], the lag time is shorter in the translational control by amber suppression (i.e. time for translation and folding) than the gene activation approaches (i.e. time for transcription, mRNA processing, translation and folding). Amber suppression also allows a more stringent control, as the background activity (if any) can be further minimised by including multiple amber codons into the gene of interest. The translational control by amber suppression approach is fully complementary to conventional genetic approaches (e.g. knockout, knockdown) that deplete a protein in cells to ‘switch off’ its function. In addition, the unnatural amino acid approach is reversible, as removing the unnatural amino acid in the growth medium will ‘switch off’ the translation of the protein of interest.

The translational switch-on process has been widely employed as a reporter system to test incorporation of new unnatural amino acids by using luminescent proteins like green fluorescent protein [46] or luciferase [20]. Upon successful incorporation, cells can emit light, whose intensity directly correlates to the unnatural amino acid incorporation efficiency. Apart from the reporter strategy, this approach has also been used to regulate function of other proteins, such as Cas9 for controllable gene editing in mouse embryos [47].

**Figure 4:**
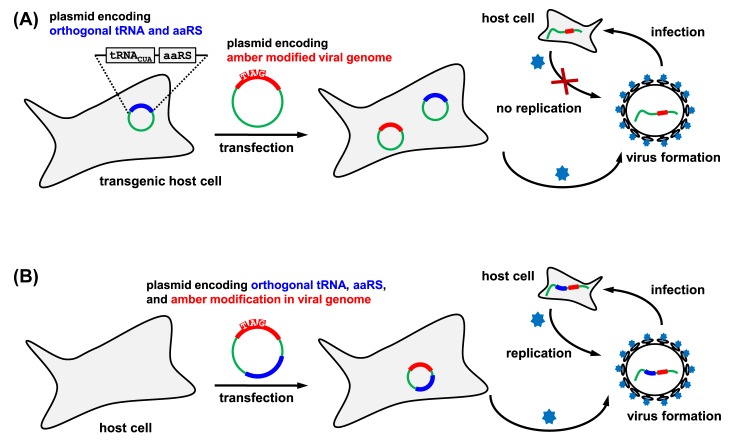
Translational activation approaches to control virus replication (**A**) Use of genetic code expansion to control replication of an amber codon tagged virus within transgenic host cells containing the orthogonal tRNA/synthetase pairs [43,48]. (**B**) Use of genetic code expansion to control replication of an amber codon tagged virus within normal host cells with the orthogonal tRNA/synthetase pair gene encoded by the viral genome [49].

Besides the general use of the ‘translational activation’ approach to study protein function, this principle has been proven to be powerful in controlling virus replication [43,48,49]. By introducing TAG codons within the virus genes, viruses can only be generated using cell lines containing an orthogonal synthetase/tRNA_CUA_ pair, and the resulting viruses are replication-incompetent in normal cells due to the lack of amber suppressor tRNA (Figure 4A) [43,48]. Such replication-incompetent viruses offer an additional tier of control for live-attenuated vaccines and significantly increase their safety. This concept has been further developed by including the genes encoding the orthogonal aaRS/tRNA pair into the viral genome (Figure 4B) [49]. In this case, viruses can be replicated in wild-type cells and the native hosts, as long as the unnatural amino acid is supplemented. Here, spatial control can also be achieved by local administration of the unnatural amino acid as demonstrated in examples of mice with an expanded genetic code [50,51]. Thus, the approach can be used for controlling viral vectors in gene therapy, where spatiotemporal virus replication and gene editing are highly desirable.

While the translational control approach is quite simple, the response is not instantaneous. There is always a lag time from when the unnatural amino acid is administered into the culture medium until the full-length protein is produced and folded. Similarly, depleting the unnatural amino acid in the growth medium will stop production of new proteins, but the protein function will only be completely switched off when all previously produced proteins are degraded in the cells. Thus, the kinetics of the switching off process largely depend on the half-life of the protein, so the response rate is the same as with genetic knockdown.

## Light-induced activation or inhibition

The slower kinetics of the translational control approach limit its applicability to study biological processes where fast response is needed. This can be addressed by using light to unmask or modify unnatural amino acids and subsequently regulate protein function. Depending on the nature of the light-responsive group, it is possible to either activate, inhibit, or reversibly switch on/off protein function (Table 2). Unnatural amino acids containing a photocage (i.e. a photolabile protecting group) [52] have been widely used for protein activation. When replacing a functionally critical amino acid residue with the corresponding photocaged amino acid, the target protein becomes inactive; upon light irradiation, the photocage is removed, thereby restoring the protein’s function. To date, photocaged cysteines (**17**–**21**), lysines (**67, 72**–**74**) and tyrosines (**102**–**105**) have been used to control enzyme function, intein splicing, protein subcellular localisation, virus–host interactions, and cell signalling cascades [13]. The light-activation approach is particularly useful for kinetic studies as it provides extreme spatiotemporal resolution. Spatial control can be achieved to even subcellular locations using focused light beams, which is virtually impossible when using the translational control approach. Theoretically, it is also possible to incorporate photocaged serine in mammalian cells as it has been demonstrated in yeast [53]. Therefore, the light-activation approach is applicable to regulate any protein that has a functionally critical cysteine, lysine, tyrosine, or serine residue in mammalian cells, including but not limited to kinases, DNA- and RNA-binding proteins, proteases, phosphatases, oxidoreductases, isomerases, and ubiquitin-modifying enzymes [54].

**Table 2:** Overview of photocaged unnatural amino acids that have been incorporated in mammalian cell systems for activating protein function upon irradiation with light of specific wavelength λ

Amino acid	Photocaging group (R)	System	Proteins	λ_decag_ (nm)
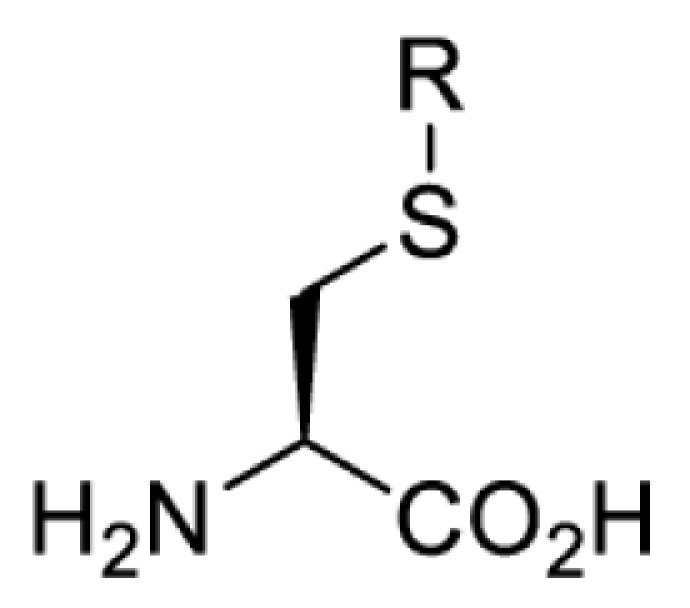	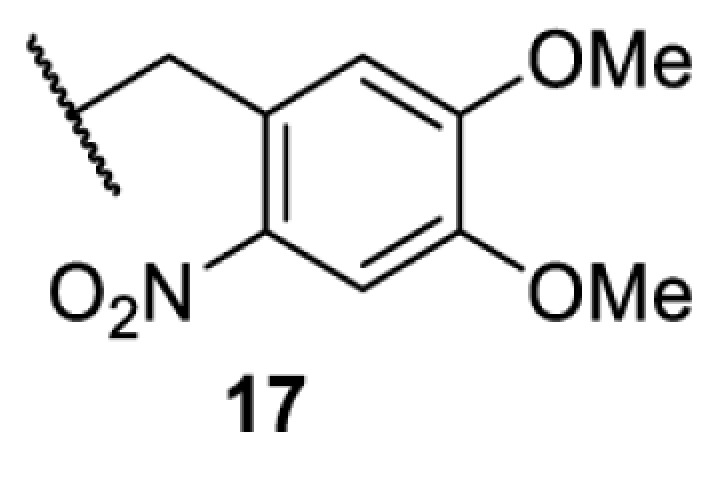	HEK293T	eGFP, potassium channel Kir2.1 [71]	385
			eGFP [72]	Long wavelength UV
	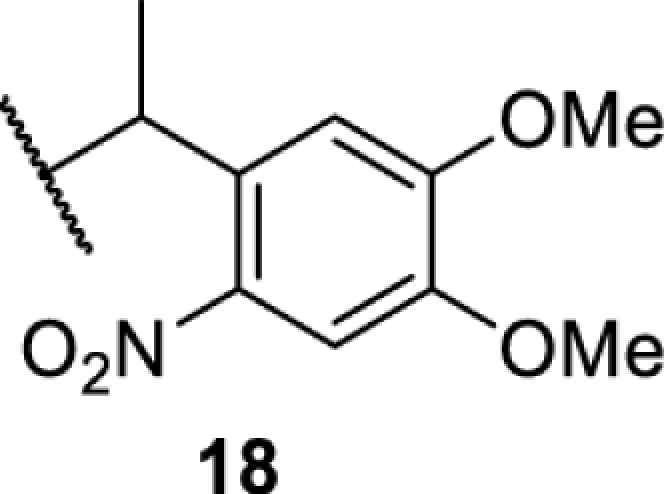		eGFP, mCherry, *Npu* DnaE intein, Src kinase [72]	
	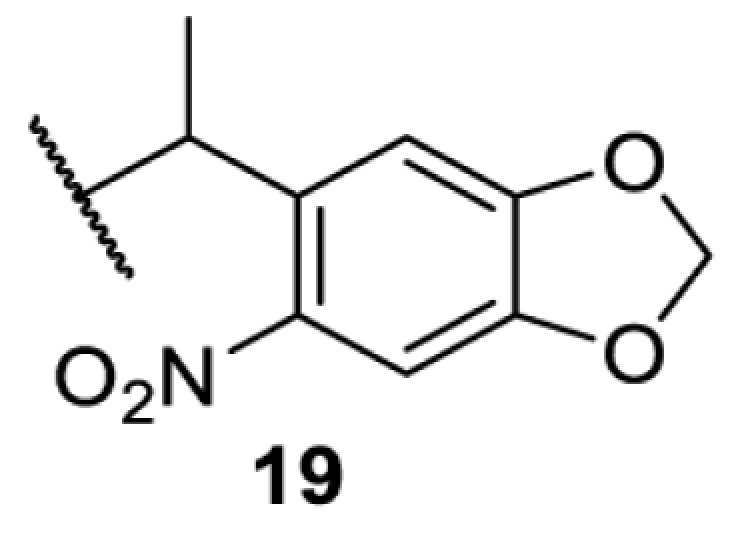		TEV protease [73,74], *Npu* DnaE intein [74]	365
	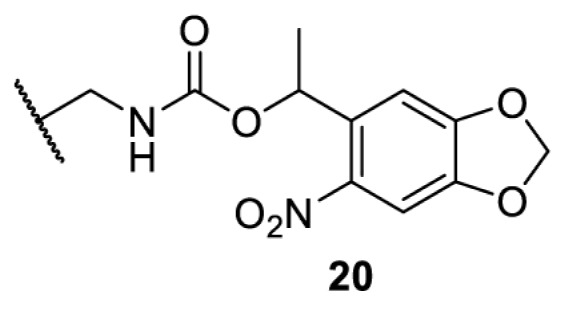		sfGFP, luciferase [75]	
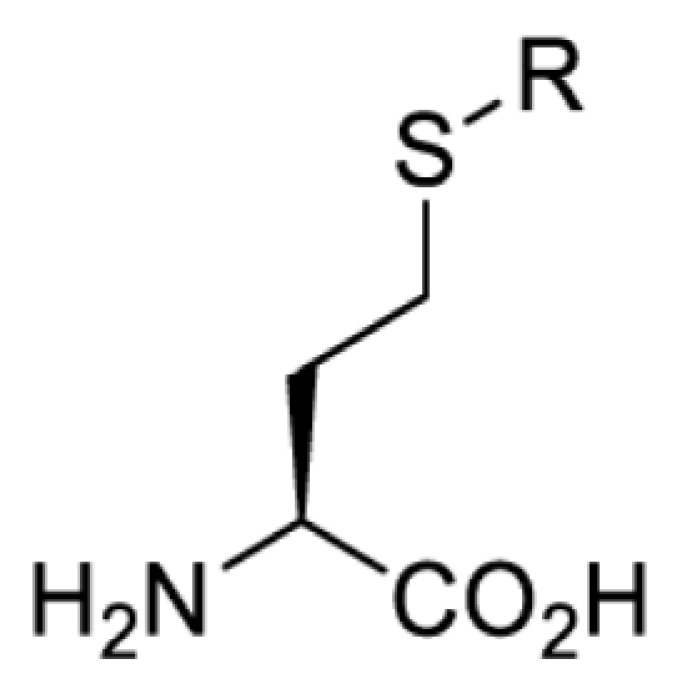	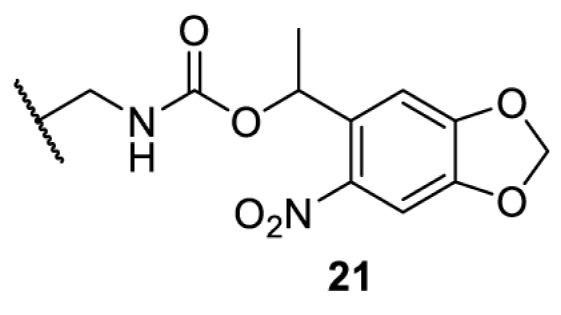	HEK 293T	sfGFP, luciferase [75]	365
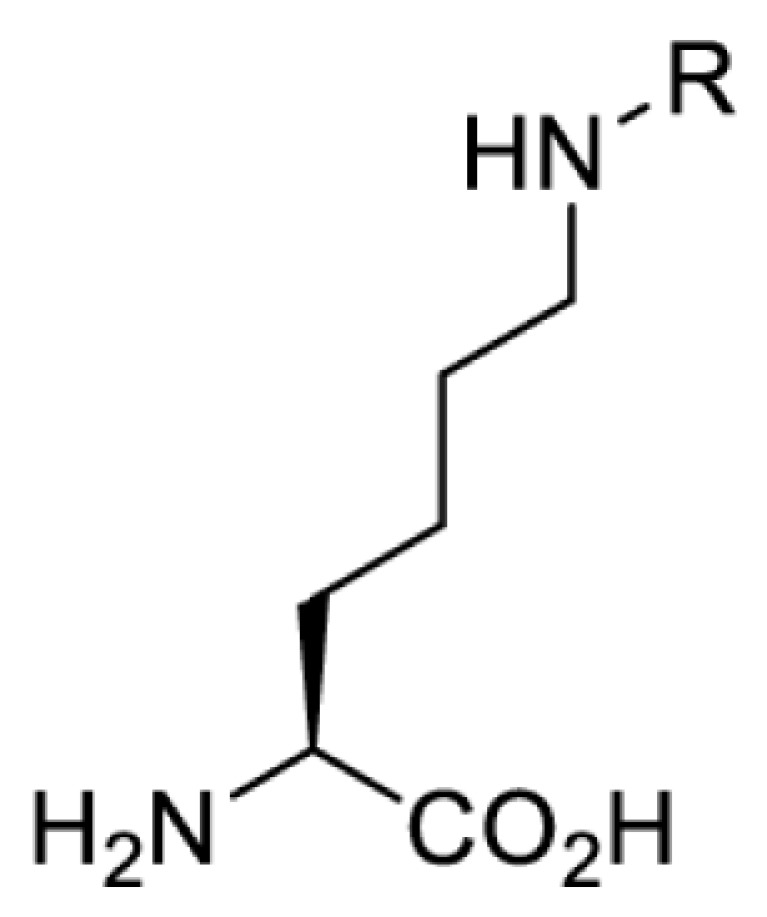	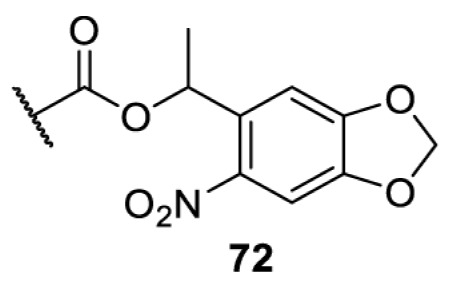	HEK293 [67,125] HEK293T [66,75,147–150,152] HEK293ET [151] HeLa [150,152]	Nuclear localisation peptide for subcellular localisation of SATB1 and FOXO3 transcription factors, and TEV protease [149]	350
			sfGFP, luciferase [75]	365
			p53 transcription factor [125]	
			Isocitrate dehydrogenase [67]	
			Cas9 endonuclease [150]	
			Cre recombinase [148]	
			Capsid of adeno-associated virus 2 [147]	
			T7RNA polymerase [152]	
			MEK1 kinase [151]	
			LCK kinase [66]	405
	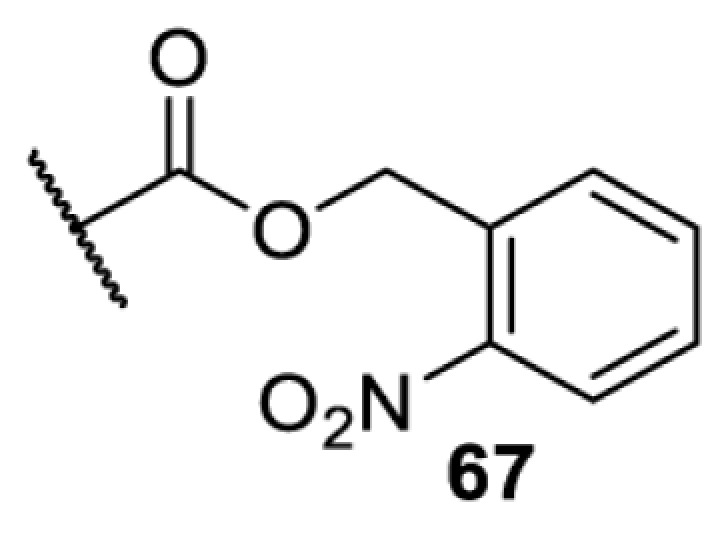	HEK293T	Luciferase [144]	365
	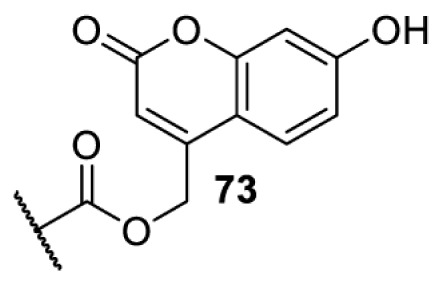	HEK293T or CHO K1	eGFP and luciferase [153]	365 405
	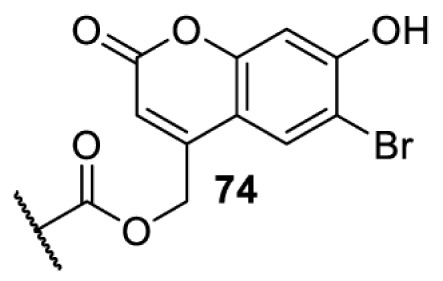			365 405 760^1^
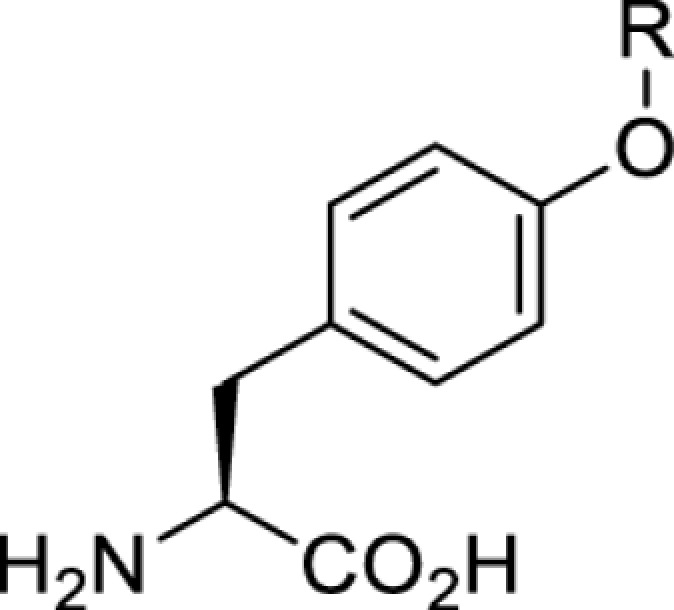	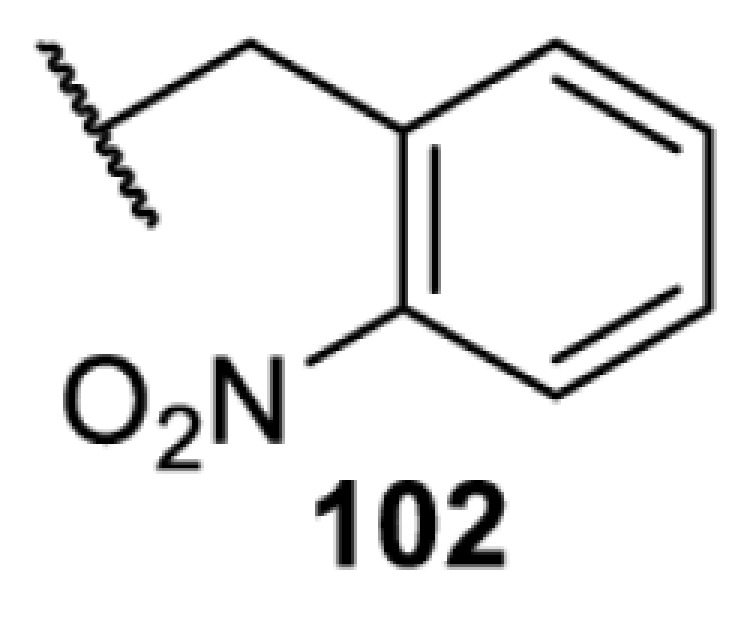	HEK293 [126] HEK293T [148,173]	Cre recombinase [148]STAT1transcription factor [126]Luciferase [173]	365
	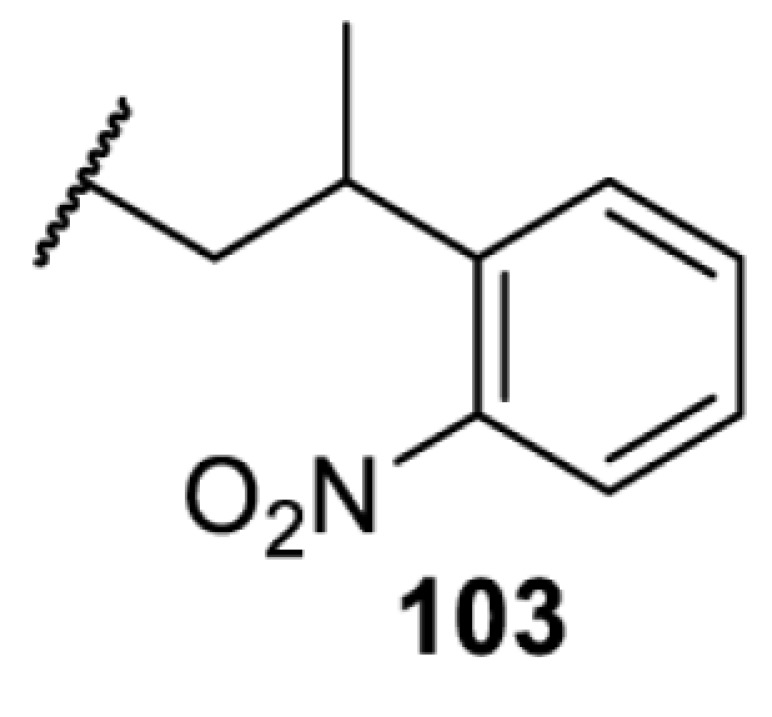	HEK293T	luciferase, TEV protease [173]	
	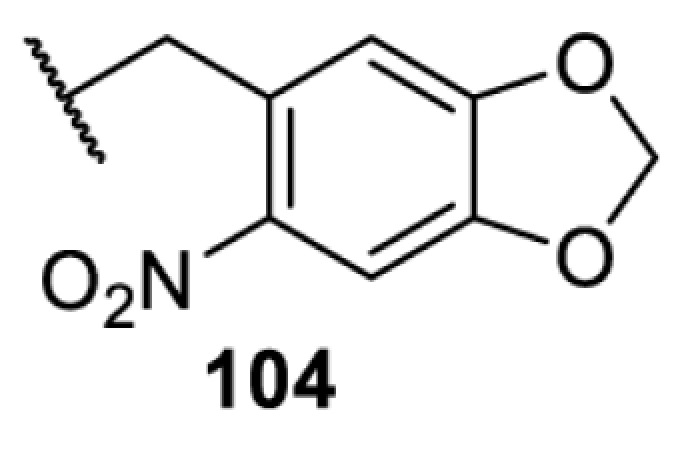			
	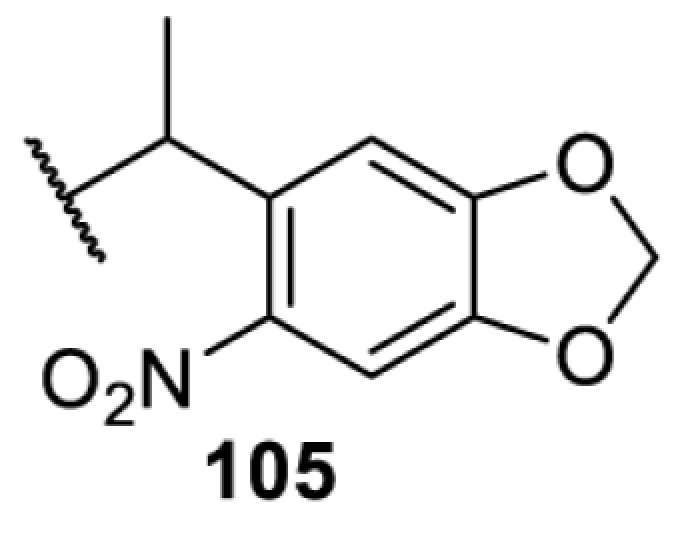			

1 Decaging is only achieved at this wavelength via a two-photon activation using a specialised multiphoton laser setup. Abbreviation: Npu, *Nostoc punctiforme*.

In contrast, the incorporation of a photocrosslinking amino acid can be used to inhibit protein function upon light irradiation [55]. In this case, a photocrosslinking amino acid is placed in the interior of the target protein. Upon light irradiation, a highly reactive functionality (e.g. radical, nitrene, carbene) is generated and reacted non-specifically with a nearby amino acid residue, causing cross-linking of the protein and subsequent abolishment of the protein’s activity. The feasibility of this approach has been demonstrated with the use of *p*-benzoylphenylalanine (**41**) in the study of glutamate receptors, GluA1 and GluA2 [55]. When compared with the use of a photocaged amino acid, inhibiting a protein by photocrosslinking does not rely on the existence of a functionally critical residue, and thus theoretically, it can be used to investigate any protein in mammalian cells. Nevertheless, for each protein target it is necessary to screen a suitable site for placing the photocrosslinking amino acid. Protein variants containing the photocrosslinking amino acid must (i) behave in the same way as the wild-type protein (i.e. phenotypically silent) before light irradiation; and (ii) be fully inhibited after light irradiation causing the photocrosslinking. Due to these criteria, the screening process can be laborious and time-consuming.

In addition to light-induced activation and inhibition, reversible regulation of a protein function can be achieved through incorporation of a photoswitchable amino acid (Table 3). For example, **48**, containing an azobenzene functionality which undergoes reversible *cis*-*trans* isomerisation upon irradiation with blue and UV light, has been used to control the activity of a glutamate receptor [56]. However, the general applicability of this approach suffers from similar constraints as inhibition by photocrosslinking. Extensive screening is often needed to identify a suitable site for incorporation, such that the resulting protein variant is fully active or inactive upon irradiation with light of a specific wavelength. At the current state of the art, there is no guarantee that such a site can be found in the target protein.

**Table 3:** Overview of photoswitchable unnatural amino acids that have been incorporated in mammalian cell systems used to modulate protein function upon irradiation with the given wavelengths λ

Amino acid	System	Proteins	λ*_trans-cis_* (nm)	λ_*cis-trans*_ (nm)
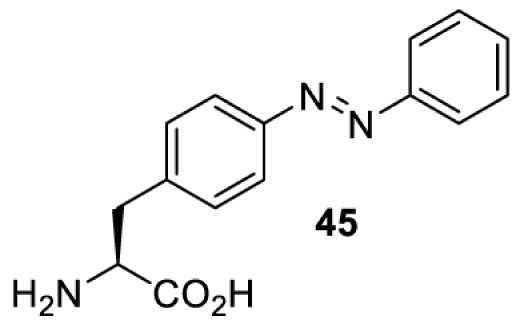	HEK 293T	Luciferase [111]	355	450
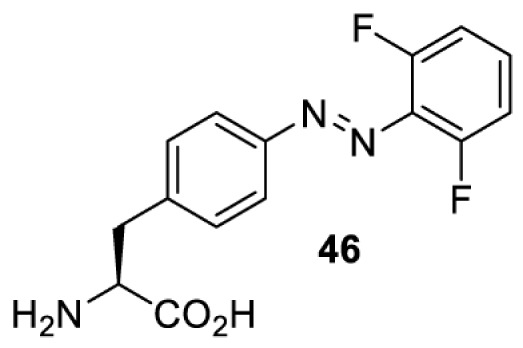			530	405
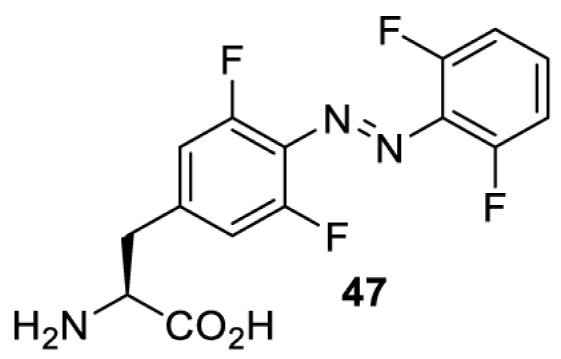				
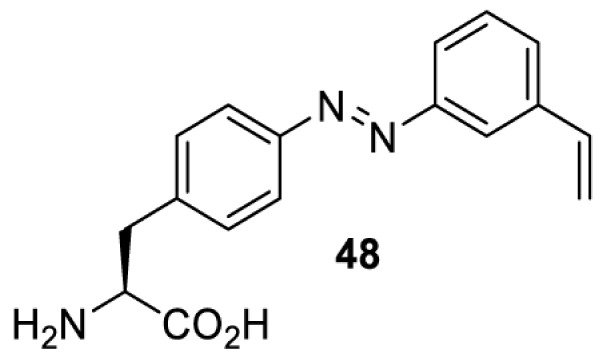		NMDAR glutamate receptor [56]	365	460

Overall, the use of light-responsive amino acids offers superior temporal control of protein function as the response is significantly faster (seconds) than the translational control by amber suppression (minutes to hours). Additionally, spatial control can be achieved at subcellular level, which is not possible with the translational approach. Generally, UV light at approximately 360 nm (i.e. UVA) is required (Tables 2 and 3) to induce the change (i.e. decaging, cross-linking, or isomerisation). However, UVA light has been shown to alter cellular signalling processes [57] or influence proper cellular function, if high intensity irradiation is applied (i.e. 50 J.cm^−2^) [58]. Though not necessarily problematic, this has to be considered when planning to apply light-responsive unnatural amino acids. Thus, there is a trend to develop new functionalities that can be modulated by light of higher wavelengths [52]. In particular, red and near-infrared light (650–750 nm) are appealing because they cause no harm to cells even under excessive exposure, and they can penetrate tissues for *in vivo* applications. To date, coumarin-caged lysines (**73** and **74**) are the only genetically incorporable unnatural amino acids that can be decaged within these wavelengths, although by two-photon approach that requires a specialised multiphoton laser setup [52]. Nevertheless, with the continuous advances in light-responsive chemical functionalities and orthogonal aaRS engineering, it is expected that more unnatural amino acids with the desired photophysical properties can be incorporated through genetic code expansion.

## Small-molecule induced activation or inhibition

In addition to light, small molecules can also be used to unmask or modify unnatural amino acids and subsequently regulate protein function with prompt response. For example, several protecting groups can be removed bioorthogonally inside live mammalian cells, and these chemistries have been used to switch on protein function by genetic code expansion. Intracellular bioorthogonal reactions that have been used in this purpose include inverse electron demand Diels–Alder reactions [18,59–61], 1,3-dipolar cycloadditions [62], Staudinger reactions [63], and palladium-catalysed propargyl removal (Table 4) [57]. Currently, all of these have only been demonstrated in caged lysine molecules (**61**, **70**, **71**, **85**) through a number of examples, including activation of luciferases, kinases, nucleases etc. Theoretically, all these protecting groups can be applied to other nucleophilic amino acids (e.g. cysteine, serine, threonine, tyrosine) subjected to successful engineering of the corresponding orthogonal synthetases.

**Table 4:** Overview of bioorthogonally protected unnatural amino acids tested in mammalian cell systems and their deprotection conditions

Amino acid		System	Proteins	Reaction	Reagent
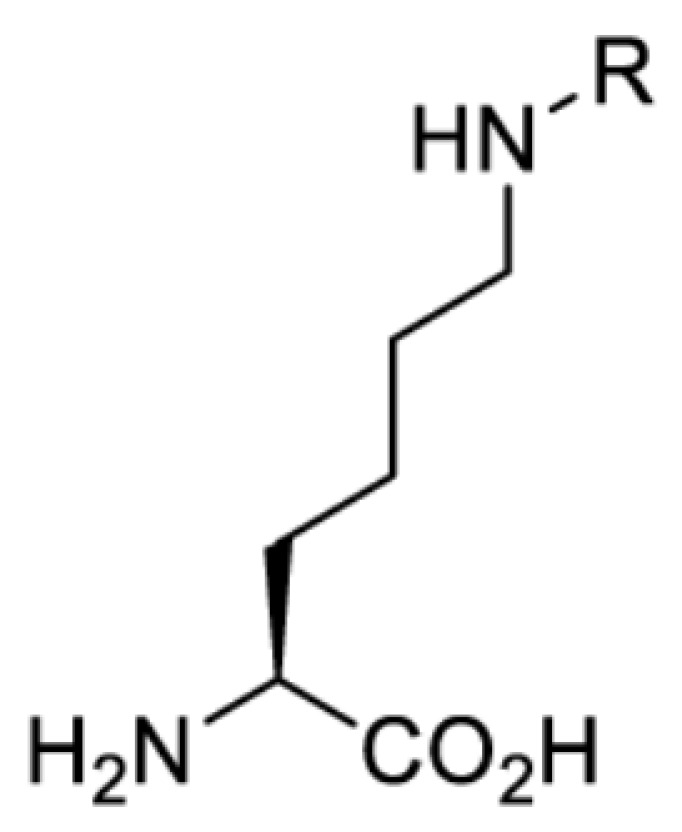	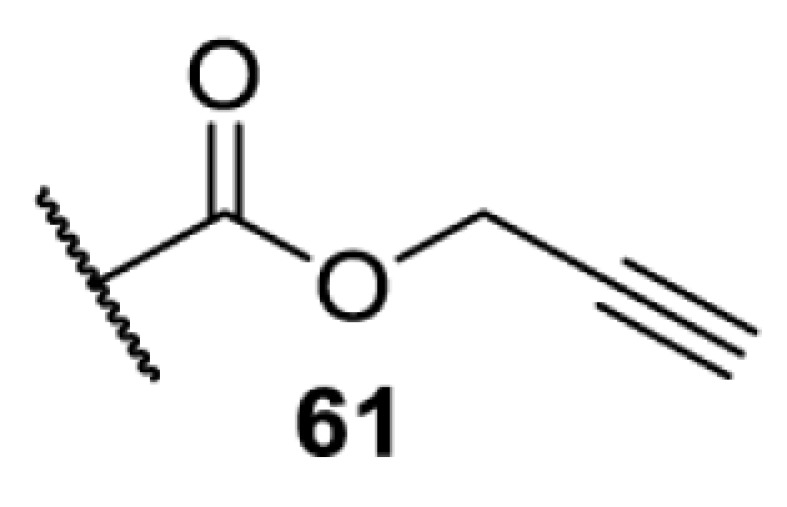	HeLa, CHO, HEK293T, IH3T3, Caco-2, A549, HeLa	GFP, OspF phosphothreonine lyase	Pd-catalysed Tsuji–Trost-like reaction	Pd(II) complexes [57]
	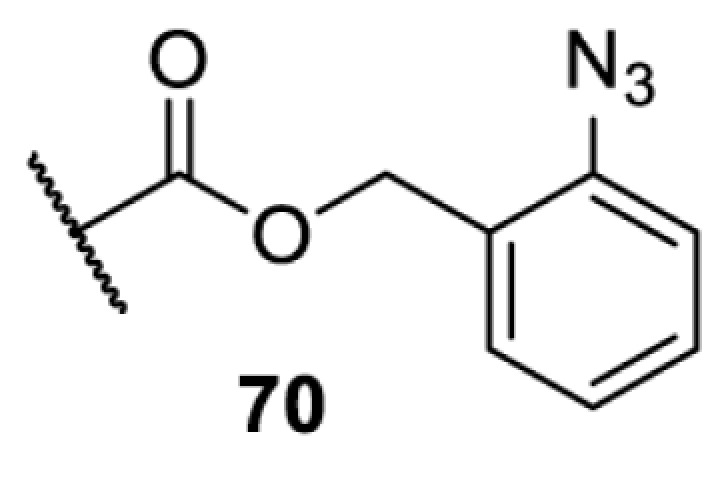	HEK293T	eGFP, SATB1 transcription factor, Cre recombinase, Cas9 endonuclease	Staudinger	Various phosphines [63]
	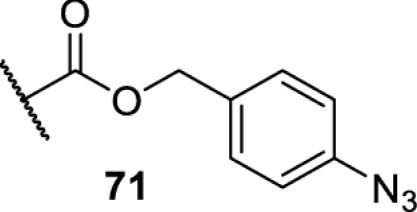	HEK293T	eGFR, luciferase, OspF phosphothreonine lyase, Src kinase	1,3-dipolar cycloaddition	*trans*-cyclooctenes [62]
	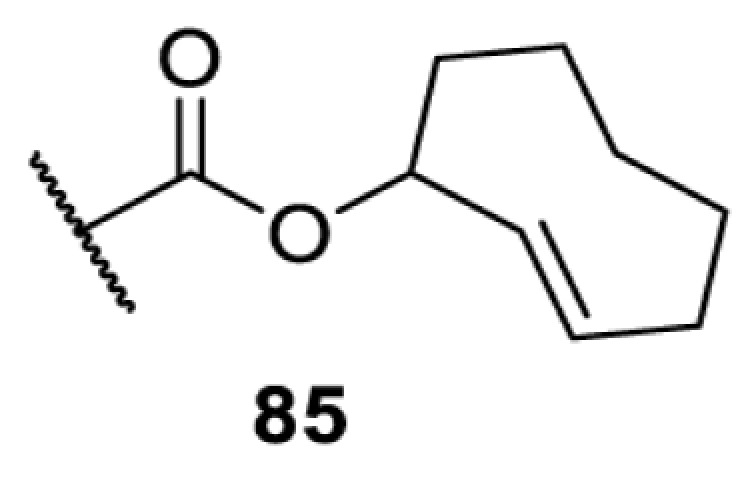	HEK293T	GFP [61], Luciferase [59,61], MEK1 [18,60] and MEK2 [18] and FAK [60] and Src [60] kinases	Inverse electron demand Diels–Alder reactions	Tetrazines [18,59–61]

**Figure 5:**
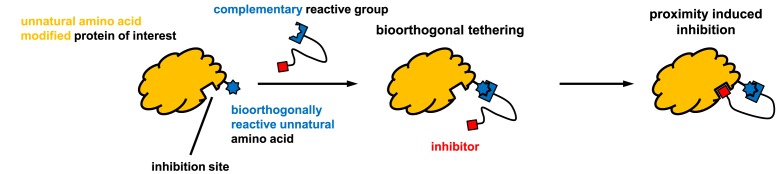
Example of the small-molecule approach to protein inhibition Use of unnatural amino acid incorporation for selective inhibition of protein function by bioorthogonal tethering [64].

On the other hand, bioorthogonal amino acids (e.g. **77** and **92**, Table 5) have been used for rapid and selective inhibition of a specific enzyme in live mammalian cells [64]. In this case, a bioorthogonal amino acid is placed into the target enzyme without affecting the enzyme function. Upon contact with an inhibitor conjugate bearing the complementary bioorthogonal group, the enzyme variant is tethered to the conjugate and thus the enzyme activity is inhibited (Figure 5). The inhibition is exquisitely selective and can even discriminate between isoforms that differ by a single amino acid residue. Using this approach, selective inhibition of an intracellular kinase for which no selective small-molecule inhibitor exists was achieved. In addition, placing a photoswitchable moiety (i.e. azobenzene) into the inhibitor conjugate enables reversible modulation of enzyme activity by light.

**Table 5:** Overview of bioorthogonally reactive unnatural amino acids that have been incorporated in mammalian cell systems used to deactivate protein function upon reaction with the specified reagents

Amino acid		System	Proteins	Reaction	Reagent
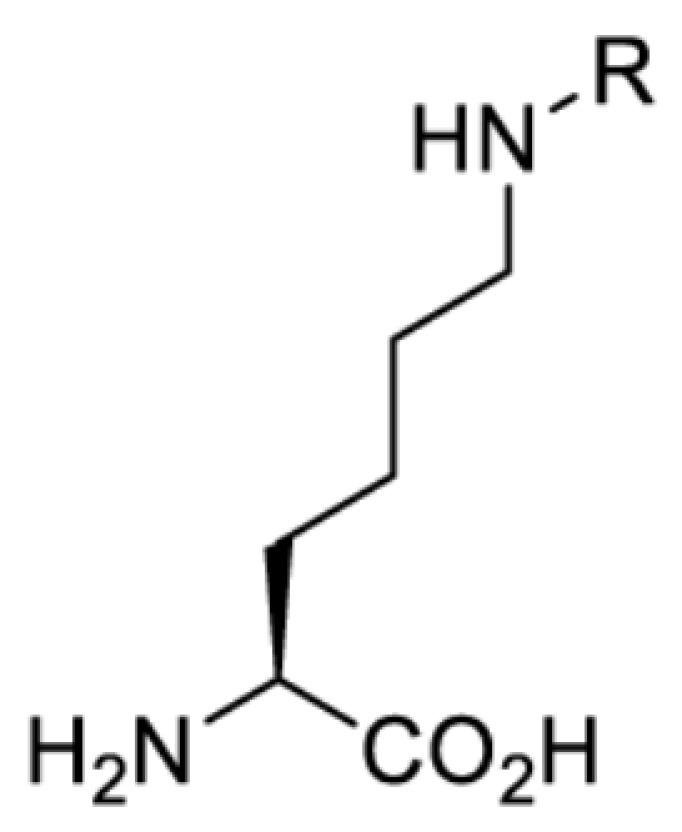	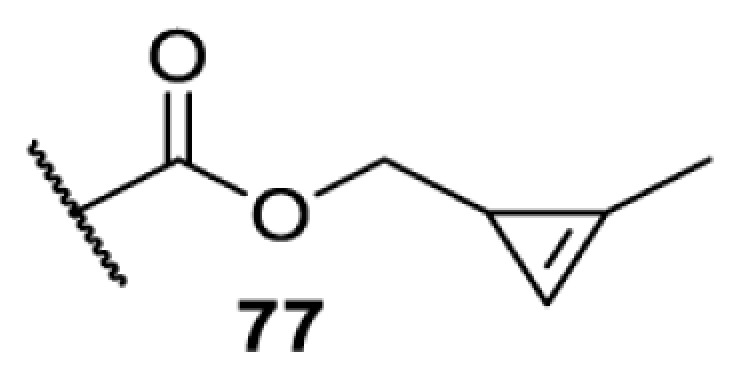	HEK293T	MEK1 and LCK kinases	inverse electron demand Diels–Alder reactions	Inhibitor–tetrazine conjugates [64]
	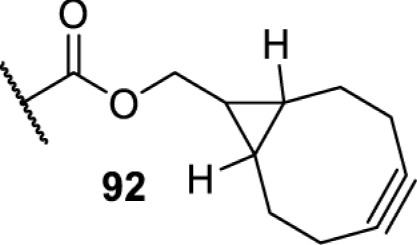		MEK1 and MEK2 kinases		

In comparison with light-induced activation or inhibition, small molecules can be used to activate or inhibit the target protein in deep animal tissue or intact animals which are not easily accessible by light. However, as mentioned above, only a few reactions have so far been shown to be bioorthogonal with high reaction rates to allow fast response [65]. The extension of this methodology is therefore tied to the development of novel bioorthogonal reactions. In contrast to the use of light for activation or inhibition, the small-molecule approach, similar to the translational approach, only allows spatial control by using cell-compartment selective compounds or reactions, or local injections.

## Conclusion

Genetic code expansion has matured into a technique that can be routinely used in mammalian systems. Controlling protein function is currently mostly achieved by translation, light, and small molecules. These methods have been summarised and discussed, and their features have been compared (Table 6). Translational control is arguably the easiest to perform but suffers from the longer response time (up to several hours). On the other hand, both light and small-molecule induced methods have a faster response (seconds to minutes). The light approach is particularly appealing where subcellular spatial resolution is needed. While all three approaches have shown promise, most of the reviewed applications are so far proof-of-principle studies. The dissemination of this technique could be enhanced by the community simplifying access to plasmids (e.g. through plasmid repository), standardising the reporting format of aaRS mutants with full sequencing information, and providing protocols with extensive details. Only general implementation of protein control by genetic code expansion in the wider scientific community to unravel new biological insights will truly demonstrate the power of these techniques [55,56,66–68]. Since genetic code expansion has also been recently demonstrated in mice [47,50,51,60,69,70], we foresee that optimisation will be tailored to target cells, tissues, and mammalian models and many of the aforementioned approaches will be applied *in vivo*, providing the complete native environment to study function of mammalian proteins. With further promotion and adaptation of genetic code expansion, this is to be expected.

**Table 6:** Comparison of protein function control approaches currently enabled by genetic code expansion

Approach	Temporal Control	Spatial control	Reversibility
Translational control	Yes, slow	Only by local administration	Yes, but high lag time
Optical control	Yes, fast	Yes, very high (to subcellular levels)	Yes, for photoswitchable amino acids
Chemical control	Yes, medium	Only by local administration	Yet to be established

## Summary

- Genetic code expansion can now be routinely used for incorporation of more than 100 different unnatural amino acids in mammalian cells using only four orthogonal aaRS/tRNA pairs and their mutants as shown in Table 1.
- Protein function can be temporally regulated (activation or inhibition) by simple translational control, i.e. supplementation of the desired unnatural amino acid to allow full-length, functional protein production.
- More rapid control can be achieved by incorporating stimuli responsive amino acids which allow activation, inhibition, or reversible regulation of protein function by light or small molecules.
- Most of the approaches have been demonstrated in proof-of-principle studies, but are ready for adaptation by the broader scientific community.

## Abbreviations

aaRS: aminoacyl-tRNA synthetase
Ec: *Escherichia coli*
Mx: *Methanomethylophilus alvus* Mx1201
PylRS: Pyrrolysyl-tRNA synthetase
